# Intracranial electrophysiology of spectrally degraded speech in the human cortex

**DOI:** 10.3389/fnhum.2023.1334742

**Published:** 2024-01-22

**Authors:** Kirill V. Nourski, Mitchell Steinschneider, Ariane E. Rhone, Joel I. Berger, Emily R. Dappen, Hiroto Kawasaki, Matthew A. Howard III

**Affiliations:** ^1^Department of Neurosurgery, The University of Iowa, Iowa City, IA, United States; ^2^Iowa Neuroscience Institute, The University of Iowa, Iowa City, IA, United States; ^3^Departments of Neurology and Neuroscience, Albert Einstein College of Medicine, Bronx, NY, United States; ^4^Pappajohn Biomedical Institute, The University of Iowa, Iowa City, IA, United States

**Keywords:** auditory cortex, cochlear implants, dorsal auditory stream, high gamma, iEEG, noise vocoder, task performance, variability

## Abstract

**Introduction:**

Cochlear implants (CIs) are the treatment of choice for severe to profound hearing loss. Variability in CI outcomes remains despite advances in technology and is attributed in part to differences in cortical processing. Studying these differences in CI users is technically challenging. Spectrally degraded stimuli presented to normal-hearing individuals approximate input to the central auditory system in CI users. This study used intracranial electroencephalography (iEEG) to investigate cortical processing of spectrally degraded speech.

**Methods:**

Participants were adult neurosurgical epilepsy patients. Stimuli were utterances /aba/ and /ada/, spectrally degraded using a noise vocoder (1–4 bands) or presented without vocoding. The stimuli were presented in a two-alternative forced choice task. Cortical activity was recorded using depth and subdural iEEG electrodes. Electrode coverage included auditory core in posteromedial Heschl’s gyrus (HGPM), superior temporal gyrus (STG), ventral and dorsal auditory-related areas, and prefrontal and sensorimotor cortex. Analysis focused on high gamma (70–150 Hz) power augmentation and alpha (8–14 Hz) suppression.

**Results:**

Chance task performance occurred with 1–2 spectral bands and was near-ceiling for clear stimuli. Performance was variable with 3–4 bands, permitting identification of good and poor performers. There was no relationship between task performance and participants demographic, audiometric, neuropsychological, or clinical profiles. Several response patterns were identified based on magnitude and differences between stimulus conditions. HGPM responded strongly to all stimuli. A preference for clear speech emerged within non-core auditory cortex. Good performers typically had strong responses to all stimuli along the dorsal stream, including posterior STG, supramarginal, and precentral gyrus; a minority of sites in STG and supramarginal gyrus had a preference for vocoded stimuli. In poor performers, responses were typically restricted to clear speech. Alpha suppression was more pronounced in good performers. In contrast, poor performers exhibited a greater involvement of posterior middle temporal gyrus when listening to clear speech.

**Discussion:**

Responses to noise-vocoded speech provide insights into potential factors underlying CI outcome variability. The results emphasize differences in the balance of neural processing along the dorsal and ventral stream between good and poor performers, identify specific cortical regions that may have diagnostic and prognostic utility, and suggest potential targets for neuromodulation-based CI rehabilitation strategies.

## Introduction

Electrical stimulation of the auditory nerve with a cochlear implant (CI) is the method of choice for treatment of severe to profound sensorineural hearing loss. Despite the tremendous progress in CI technology, including hardware and processing strategies, there remains a considerable variability in speech perception outcomes following implantation (Geers, 2006; Pisoni et al., 2017; Carlyon and Goehring, 2021). Multiple variables have been implicated to contribute to this variability, including age of onset, duration, and severity of hearing loss, as well as co-morbidities such as neurodevelopmental delays (Birman and Sanli, 2020; Sharma et al., 2020; Shinagawa et al., 2023). Recently, the function and plasticity of central auditory pathways have been emphasized as major contributing factors to the variability in speech perception outcomes (Moberly et al., 2016; Glennon et al., 2020; Pavani and Bottari, 2022).

Assessing auditory processing at the cortical level in CI users is methodologically difficult. The presence of implanted hardware precludes fMRI and MEG studies, while the electrical stimulus artifact generated by the CI represents a challenge for scalp EEG recordings (Intartaglia et al., 2022). Due to these limitations, positron emission tomography and, more recently, functional near-infrared spectroscopy have become the dominant methods to assess cortical function in CI users (e.g., Saliba et al., 2016; Yoshida et al., 2017; Han et al., 2019). While these methods provide insight into cortical physiology associated with clinical outcomes (Levin et al., 2022; Zhou et al., 2023), both methods have limitations in their spatiotemporal resolution. These limitations hinder detailed analyses of the topography and time course of cortical activation in response to CI stimulation.

Intracranial electroencephalography (iEEG) in patients undergoing chronic invasive monitoring for pharmacologically resistant epilepsy is the gold standard for assessing cortical function in humans (Crone et al., 2006; Chang, 2015; Nourski and Howard, 2015; Sonoda et al., 2022). Rhone et al. (2012), Nourski et al. (2013b), and Miller et al. (2021) had the opportunity to use iEEG to study a post-lingually deaf patient who was a successful CI user. Using a variety of experimental paradigms, these studies established the feasibility of recording iEEG auditory responses to CI stimulation and showed that basic auditory cortex response properties in a CI user with over 20 years of experience were comparable with those seen in hearing individuals. These types of case studies are obviously rare and therefore other experimental approaches need to be utilized to better understand cortical function in CI users.

One such approach is the use of spectrally degraded acoustic stimuli presented to normal-hearing individuals. Stimuli of this type approximate sensory input to the central auditory system in CI users (Merzenich, 1983; Shannon et al., 1995). Spectral degradation is exemplified by a noise vocoder, wherein speech is parceled into several frequency bands. The resultant temporal envelopes are used to modulate bandpass noise carriers. The number of frequency bands affects spectral complexity and resultant intelligibility akin to the number of independent stimulation channels in a CI (Davis et al., 2005). Normal hearing listeners exhibit variability in their perception of noise vocoded speech, reminiscent of the variability in clinical outcomes in CI users (Scott et al., 2006). Non-invasive studies have related this variability to several putative biomarkers, including the extent of activation in non-primary auditory cortex (Scott et al., 2006; Lawrence et al., 2018) and left inferior frontal gyrus (Lawrence et al., 2018).

To date, several iEEG studies utilized noise-vocoded speech (Billig et al., 2019; Nourski et al., 2019; Xu et al., 2023), mainly focusing on activation within the high gamma iEEG band (∼70–150 Hz) and suppression in the alpha band (∼8–14 Hz). High gamma responses to vocoded speech in non-core auditory cortex were found to differ with respect to spectral complexity and intelligibility to a greater degree than in core auditory cortex (posteromedial portion of Heschl’s gyrus, HGPM) (Nourski et al., 2019). Xu et al. (2023) demonstrated robust tracking of vocoded speech by high gamma power within core auditory cortex. High gamma responses to noise-vocoded stimuli on the lateral STG were found to be associated with better speech recognition performance (Nourski et al., 2019). Alpha suppression in non-core auditory cortex in the anterolateral portion of Heschl’s gyrus (HGAL) was greater for clear than vocoded speech (Billig et al., 2019).

While these iEEG studies have brought new insights into the processing of spectrally degraded speech at the level of canonical auditory cortex, the degree of activation along the dorsal and ventral auditory cortical processing streams remains an area of active investigation. Speech perception engages a wide array of parallel and serial circuits that begin at the level of the auditory cortex (Hamilton et al., 2021; Banks et al., 2023) and extend into sensory, cognitive, and behavioral networks (Herbet and Duffau, 2020). The dorsal processing stream extends from the posterior portion of the superior temporal gyrus (STGP) into prefrontal cortex via parietal cortex including supramarginal gyrus (SMG) as well as the precentral gyrus (PreCG). Non-invasive studies have specifically implicated this processing stream as relevant to speech perception under challenging listening conditions (Wong et al., 2008; Murakami et al., 2012; Elmer et al., 2017). In hearing-impaired individuals, higher-order areas are often recruited for additional basic sound processing, potentially decreasing the resources available for other cognitive functions (Gao et al., 2023).

The current study leveraged the superior spatiotemporal resolution of intracranial electroencephalography (iEEG) to study processing of spectrally degraded speech across multiple levels of the cortical hierarchy, as envisioned by current models (Hickok and Poeppel, 2007; Rauschecker and Scott, 2009; Chang et al., 2015; Herbet and Duffau, 2020; Banks et al., 2023) and supported by response onset latency measurements (Hamilton et al., 2021; Nourski et al., 2021, 2022). Taking advantage of extensive electrode coverage across multiple participants, this study examined whether hemispheric asymmetries were present in responses to spectrally degraded speech and investigated relationships between cortical activity patterns and the accuracy of speech perception.

The study revisited the experimental paradigm previously used in Nourski et al. (2019) with a more expansive analysis of speech processing at the phonemic level. The task involved discriminating between two consonants that varied in place of articulation (/b/ and /d/) to characterize speech perception as a prerequisite for more complex aspects of speech comprehension, including lexical and semantic processing. Discrimination of stop consonants based on place of articulation was chosen over voicing or manner, as they are more easily discriminated with limited spectral information (Shannon et al., 1995). By contrast, place of articulation of stop consonants, given its spectral nature, is a highly relevant feature when using vocoded speech as a model for CI speech perception. Indeed, CI users often experience difficulty with this speech contrast over others (McKay and McDermott, 1993; Skinner et al., 1999; Vandali, 2001; Välimaa et al., 2002; Winn and Litovsky, 2015). Furthermore, research has shown that variability in perception of place of articulation was a clear distinguishing feature between overall better and poorer performing CI listeners (Munson et al., 2003).

The present study sought to investigate involvement of the dorsal and ventral pathways in addition to canonical auditory cortex. Emphasis was placed on the analysis of both high gamma activation and alpha suppression. This approach measures feedforward processing and release from feedback inhibition by higher-order areas, respectively (Fontolan et al., 2014), as well as facilitating comparison with non-invasive studies. Focus on alpha suppression was motivated by the contribution of top-down cognitive and linguistic processing to CI outcomes (Moberly et al., 2016; Moberly, 2020) and the more general relevance of cortical alpha activity in modulating sensory responses (Billig et al., 2019; Bastos et al., 2020). Previous iEEG studies demonstrated that greater alpha suppression in auditory cortex was associated with better performance in auditory tasks (Nourski et al., 2021, 2022). Using this approach, the current study identified differences in high gamma augmentation and alpha suppression along the dorsal and ventral pathways that may serve as useful biomarkers to aid evaluation of post-implantation rehabilitation strategies in CI users.

## Materials and methods

### Participants

Participants were 15 neurosurgical patients (6 female, age 18–51 years old, median age 33 years old) diagnosed with medically refractory epilepsy who were undergoing chronic iEEG monitoring to identify potentially resectable seizure foci. Ten of these participants had previously been reported in a more limited study that focused on high gamma activity within canonical auditory cortex (Nourski et al., 2019). Demographic, iEEG electrode coverage, and seizure focus data for each participant are presented in Table 1. The prefix of the participant code indicates the hemisphere with predominant electrode coverage (L for left, R for right, B for bilateral). Seven participants out of 15 had electrode coverage in both hemispheres. Three participants had predominant electrode coverage in the left hemisphere, three had predominant coverage in the right hemisphere, and one (B335) had comparable coverage in both. All 15 participants were right-handed. Nine participants were left hemisphere language dominant per Wada testing, two (R263 and R672) had bilateral language dominance. In the four remaining participants (R250, R316, R320, R322), Wada test was not performed, and left language dominance was assumed.

**TABLE 1:** Participant demographics, electrode coverage and task performance.

Participant^1^	Demographics			Electrode coverage		Seizure focus
	Age (years)	Sex^2^	Language dominance^1^	Subdural arrays^3^	*n*_*sites*_ ^4^	
L222*	33	M	L	Y	179	L medial temporal
L237*	27	M	L	Y	168	L anterior, medial, ventral temporal
R250	50	F	L	Y	43	R lateral frontal
L258	38	M	L	Y	176	Broad, including L temporal
R263*	33	F	B	Y	97	R inferior lateral frontal, posterior ventral frontal, anterior insula
L275*	30	M	L	Y	218	L lateral ventral temporal
L282*	41	M	L	N	17	L posterior superior temporal, ventral frontal
R288*	20	M	L	Y	203	R temporal pole, posterior suprasylvian cortex
L307*	31	F	L	Y	95	L posterior insula
R316*	51	F	L	Y	230	R medial temporal
R320*	29	F	L	Y	78	R medial temporal
R322	33	M	L	Y	144	R posterior lateral frontal
B335*	36	F	L	Y	85	Bilateral medial temporal
R672	22	M	B	N	110	Undetermined
L702	33	M	L	N	179	Multifocal, including L temporal

^1^L: left; R: right; B: bilateral.

^2^F: female; R: male.

^3^Y: yes; N: no.

^4^Number of recording sites examined in the present study (excluding recording sites identified as seizure foci or characterized by excessive noise and depth electrode contacts in white matter or outside the brain).

*Previously studied in Nourski et al. (2019).

All participants were native English speakers except for L275, a 30-year-old native Bosnian speaker with 2 years of English formal education and 13 years of exposure. All participants except R250 had pure-tone average thresholds (PTA_0_._5–2_; at 500, 1,000, 2,000 Hz) and speech reception thresholds (SRTs) within 20 dB HL (left/right average). Participant R250 had a PTA_0_._5–2_ threshold of 38.3 dB HL and SRT of 30 dB. All participants except L275 had the left-right average word recognition scores (WRSs) of 96% or higher. Participant L275 had a WRS of 92%.

Pre-operative neuropsychological evaluation (Wechsler Adult Intelligence Scale 4th Edition) included full-scale intelligence quotient (FSIQ) and verbal comprehension index (VCI) assessments in all participants except R263 and L282. Working memory index (WMI) assessment in all participants. Median FSIQ, VCI and WMI scores were 83 (range 58–117), 87 (range 58–112), and 86 (range 63–122), respectively. The lowest scores were in participant L275 and were in part attributed by the neuropsychologist to his relatively limited exposure to the English language.

Research protocols were approved by the University of Iowa Institutional Review Board and the National Institutes of Health. Written informed consent was obtained from all participants. Research participation did not interfere with acquisition of clinical data, and participants could rescind consent at any time without interrupting their clinical evaluation.

### Stimuli and procedure

Experimental stimuli were the utterances /aba/ and /ada/, spoken by a male talker (Supplementary Figure 1). The stimuli were taken from The Iowa Audiovisual Speech Perception Laser Video Disc (Tyler et al., 1989). They were spectrally degraded using a noise vocoder with 1, 2, 3 or 4 frequency bands following the approach of Shannon et al. (1995) using a modification of Dr. Chris Darwin’s Shannon script,^1^ implemented in Praat v.5.2.03 environment (Boersma, 2001). The four noise vocoders were defined as follows:

1-band: 0–4,000 Hz;

2-band: 0–1,500 Hz, 1,500–4,000 Hz;

3-band: 0–800 Hz, 800–1,500 Hz, 1,500–4,000 Hz;

4-band: 0–800 Hz, 800–1,500 Hz, 1,500–2,500 Hz, 2,500–4,000 Hz.

Temporal envelopes of the vocoded stimuli were low-pass filtered at 50 Hz. All stimuli, including the original (clear) utterances, were low-pass filtered at 4 kHz. The duration of /aba/ and /ada/ was 497 and 467 ms, respectively, with the onset of the consonant at 232 and 238 ms, respectively. The utterance /aba/ steady-state vowel fundamental frequencies (F0) of 136 and 137 Hz for the first and the second /a/, respectively, while /ada/ had steady-state vowel fundamental frequencies of 130 and 112 Hz for the first and the second /a/, respectively.

Experiments were carried out in a dedicated, electrically-shielded suite in The University of Iowa Clinical Research Unit. The room was quiet, with lights dimmed. Participants were awake and reclining in a hospital bed or an armchair. The stimuli were delivered at a comfortable level (typically 60–65 dB SPL) via insert earphones (Etymotic ER4B, Etymotic Research, Elk Grove Village, IL, USA) coupled to custom-fit earmolds. Stimulus delivery was controlled using Presentation software (Version 16.5; Neurobehavioral Systems).

Stimuli (40 repetitions of each) were presented in random order in a two-alternative forced choice (2AFC) task. Participants were instructed to report whether they heard an /aba/ or an /ada/ following each trial by pressing one of the two buttons on a Microsoft Sidewinder video game controller or a USB numeric keypad: left for /aba/ and right for /ada/. The two choices were shown to the participant on a computer screen, and 250 ms following the participant’s button press the correct answer was highlighted for 250 ms to provide real-time feedback on the task performance. The next trial was presented following a delay of 750–760 ms. The self-paced task took between 15 and 29 min. to complete (median duration 20 min). The real-time feedback was provided to help motivate the participants to complete the task. Preliminary studies indicated that participants found lack of feedback over the course of the task frustrating, which could negatively affect their willingness to continue participation in this and other research tasks.

### Recordings

Recordings were obtained using either subdural and depth electrodes, or depth electrodes alone. Implantation plans were based on clinical requirements, as determined by a team of epileptologists and neurosurgeons. Details of electrode implantation, recording and iEEG data analysis have been described previously (Nourski and Howard, 2015). Electrode arrays were manufactured by Ad-Tech Medical (Racine, WI). Subdural arrays, implanted in 12 participants out of 15, consisted of Pt/Ir discs (2.3 mm diameter, 5–10 mm inter-electrode center-to-center distance), embedded in a silicon membrane. Stereotactically implanted depth arrays included between 4 and 12 cylindrical contacts along the electrode shaft, with 5–10 mm inter-electrode distance. A subgaleal electrode, placed over the cranial vertex near midline, was used as a reference in all participants.

Data acquisition was controlled by a TDT RZ2 real-time processor (Tucker-Davis Technologies, Alachua, FL) in participants L222 through B335 and by a Neuralynx Atlas System (Neuralynx, Bozeman, MT) in participants R672 and L702.

Recorded data were amplified, filtered (0.7–800 Hz bandpass, 5 dB/octave rolloff for TDT-recorded data; 0.1–500 Hz bandpass, 12 dB/octave rolloff for Neuralynx-recorded data), digitized at a sampling rate of 2034.5 Hz (TDT) or 2,000 Hz (Neuralynx) and downsampled to 1,000 Hz for analysis.

### Analysis

Participants’ performance in the behavioral task was characterized in terms of accuracy (% of correct responses), sensitivity (*d’*) and reaction times (RT). For each of the five conditions (1-, 2-, 3-, 4-band and clear), statistical significance of across-participant median task accuracy relative to chance performance was established using one-tailed Wilcoxon signed rank tests. Differences in task accuracy and *d’* between adjacent conditions were established using two-tailed Wilcoxon signed-rank tests. For RTs, pairwise comparisons between adjacent stimulus conditions were performed using linear mixed effects model analysis. Stimulus condition and participant were modeled as categorical fixed effect and a random intercept, respectively.

Participants were rank-ordered according to their average accuracy in the 3- and 4-band conditions (3&4-band accuracy) to examine the relationships between participants’ demographic, audiometric, neuropsychological and clinical background, and task performance. Effects of participants’ demographic background – sex and age – were examined using a two-tailed Wilcoxon signed-rank test and Spearman’s rank order correlation, respectively. To investigate relationships between the participants’ peripheral auditory function and task performance, 0.5, 1, and 2 kHz pure-tone average (PTA_0_._5–2_) thresholds were computed. Left-right averages were correlated with 3&4-band accuracy using Spearman’s rank order correlation. Relationships between participants’ speech recognition thresholds (SRT), word recognition scores (WRS), and their task performance were investigated in an analogous manner. To test whether neuropsychological and clinical background could account for the performance variability, neuropsychological assessment indices (FSIQ, VCI, WMI), epilepsy age of onset, and duration of the seizure disorder were correlated with 3&4-band accuracy using Spearman’s rank order correlation. Finally, the effect of seizure focus localization to the left or right hemisphere on task performance was examined using a two-tailed Wilcoxon signed-rank test.

Anatomical localization of recording sites was based on pre- and post-implantation structural MRI and computed tomography (CT) data and, for subdural arrays, aided by intraoperative photography. Images were initially aligned with pre-operative T1 MRI scans using linear coregistration implemented in FSL (FLIRT) (Jenkinson et al., 2002). Accuracy of electrode localization within the pre-operative MRI space was refined using three-dimensional non-linear thin-plate spline warping (Rohr et al., 2001).

Recording sites identified as seizure foci or characterized by excessive noise, and depth electrode contacts localized outside cortical gray matter, were excluded from analyses. In total, 2,051 recording sites were studied across the 15 participants. Each recording site was assigned to one of 50 regions of interest (ROIs) based on anatomical reconstructions of electrode locations in each participant (Supplementary Figure 2). ROI assignment was based on automated cortical parcellation as implemented in the FreeSurfer software package (Destrieux et al., 2010, 2017) and refined based on visual inspection of anatomical reconstruction data. For recording sites in Heschl’s gyrus, delineation of the border between core auditory cortex and adjacent non-core areas [posteromedial (HGPM) and anterolateral (HGAL) portions, respectively] was performed in each participant using multiple physiological criteria. These included phase-locked responses to click trains and discontinuities in the morphology of the averaged evoked potential waveforms (Brugge et al., 2009; Nourski et al., 2016). STG was subdivided into posterior and middle non-core auditory cortex ROIs (STGP and STGM), and anterior auditory-related ROI (STGA) using the transverse temporal sulcus and ascending ramus of the Sylvian fissure as anatomical boundaries. Middle and inferior temporal gyrus were each divided into posterior, middle, and anterior ROIs (MTGP, MTGM, MTGA, ITGP, ITGM, ITGA) by dividing each gyrus into three approximately equal-length thirds. Angular gyrus was divided into posterior (AGP) and anterior (AGA) ROIs along the angular sulcus.

Cortical activity elicited by the speech stimuli was measured and characterized as event-related band power (ERBP). For each recording site, trials with voltage deflections exceeding five standard deviations from the mean calculated over the entire duration of the recording were assumed to be artifact and rejected. Time-frequency analysis was carried out using a demodulated band transform method (Kovach and Gander, 2016).^2^ The power of the iEEG signal was computed within overlapping frequency windows of variable bandwidth for theta (center frequencies 4–8 Hz, 1 Hz step), alpha (8–14 Hz, 2 Hz step), beta (14–30 Hz, 4 Hz step), low gamma (30–70 Hz, 10 Hz step) and high gamma (70–150 Hz, 20 Hz step) iEEG bands. For each center frequency, power was log-transformed, segmented into single trial epochs, normalized by subtracting the mean log power within a reference interval (100–200 ms before stimulus onset in each trial), and averaged over trials to obtain ERBP for each center frequency.

Quantitative analysis of iEEG data focused on high gamma and alpha ERBP, calculated by averaging power envelopes for center frequencies between 70–150 and 8–14 Hz, respectively. Mean high gamma and alpha ERBP values were computed within 250–500 ms and 500–750 ms windows relative to stimulus onsets, respectively. The 250–500 ms time interval corresponded to the approximate duration of the second syllable of the stimuli. ERBP was then averaged across trials for each of the three levels of spectral degradation (1- and 2-band combined, 3- and 4-band combined, and clear speech), pooled over /aba/ and /ada/ trials.

Correction for multiple comparisons was performed using the false discovery rate (FDR) approach (Benjamini and Hochberg, 1995). At the single-site level, significance of high gamma augmentation and alpha suppression for the three levels of spectral degradation was established using one-sample one-tailed *t*-tests (significance threshold *p* = 0.05, FDR-corrected). Differences between adjacent levels of spectral degradation (i.e., 1&2-band vs. 3&4-band and 3&4-band vs. clear) were examined using two-sample two-tailed *t*-tests (significance threshold *p* = 0.05, FDR-corrected). Eight patterns of high gamma augmentation were defined as follows (shown schematically in Figure 4A):

1. Spectral complexity: significant ERBP increase from 1&2 to 3&4 and from 3&4 to clear.
2. Potential intelligibility: significant increase from 1&2 to 3&4 and no significant difference between 3&4 and clear.
3. Clear-preferred: no significant difference between 1&2 and 3&4, significant response to 3&4, and significant increase from 3&4 to clear.
4. Clear-specific: no significant difference between 1&2 and 3&4, no significant response to 3&4, and significant increase from 3&4 to clear.
5. Vocoded-preferred: no significant difference between 1&2 and 3&4, significant response to 3&4, and significant decrease from 3&4 to clear.
6. Non-selective: no significant difference between 1&2 and 3&4, no significant difference between 3&4 and clear, and significant responses to all three levels of spectral degradation.
7. Weak: no significant difference between 1&2 and 3&4, no significant difference between 3&4 and clear, and significant responses to one or two out of three levels of spectral degradation.
8. No response: no significant response to any of the three levels of spectral degradation.

Likewise, the same eight patterns were defined for alpha ERBP, albeit based on suppression rather than augmentation. None of the sites exhibited what could have been interpreted as a “3&4-preferred” high gamma or alpha pattern (i.e., a significant response to 3&4 that is significantly greater than 1&2 and clear).

To visually summarize data across multiple participants, locations of recording sites were plotted in MNI coordinate space, color-coded by response pattern, and projected onto the FreeSurfer average template brain for spatial reference. Distribution of responsive sites within precentral gyrus (PreCG) was also examined with respect to motor somatotopy by depicting the barycenters of hand, wrist, fingers, lips, tongue, and larynx regions and their standard deviations as reported by Roux et al. (2020).

To relate task performance to patterns of cortical activity, average accuracy between the 3 and 4 band conditions in each participant was used to characterize participants’ performance as either good (78.8–91.3% accuracy), intermediate (66.3–70.6%) or poor (47.5–62.5%), corresponding to the three tertiles of the 3&4-band accuracy distribution. Distributions of response patterns were compared on ROI level between left and right hemisphere and between good and poor performers using Fisher exact tests with the Freeman–Halton extension (Freeman and Halton, 1951), implemented with fisher.test in R (R Core Team),^3^ and corrected for multiple comparisons with FDR correction. ROI-level comparisons were performed for ROIs with > 10 recording sites from at least 2 participants in each comparison group (i.e., left and right, or good and poor). Relationships between the magnitude of participants’ cortical responses to vocoded and clear stimuli and their average accuracy in the 3&4-band condition were examined using Spearman’s rank correlation tests with FDR correction for multiple comparisons.

## Results

### Behavioral task performance

**FIGURE 1:**
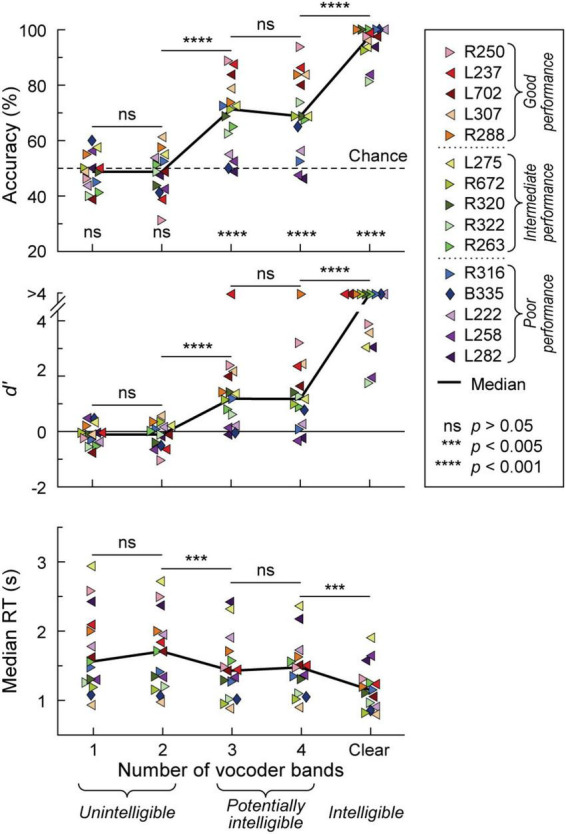
Behavioral 2AFC task performance. Accuracy, sensitivity (*d*’) and median reaction times (RTs) are plotted in the top, middle, and bottom panels, respectively for vocoded (1–4 bands) and clear stimuli. Data from the 15 participants and across-participant median values are plotted as symbols and solid lines, respectively. Left- and right-pointing arrowhead symbols represent participants with mainly left- and right hemisphere electrode coverage, respectively. Participant B335 with bilateral electrode coverage is denoted by a diamond. Significance (Wilcoxon signed rank tests) of across-participant median task accuracy relative to chance (dotted line) is indicated in the top panel. Pairwise comparisons between adjacent stimulus conditions across participants were performed using Wilcoxon rank-sum tests for accuracy and *d’*, and linear mixed effects models for RTs. In the figure legend, participants are rank-ordered based on their 3&4-band accuracy, from best (R250) to worst (L282). Dotted lines in the figure legend correspond to the tertile intervals used to differentiate good, intermediate, and poor task performance.

Participants’ task performance was characterized in terms of accuracy (% correct responses), sensitivity (*d’*) and reaction time (RT). All participants exhibited chance performance (accuracy ∼50%, *d’* ∼0) in the 1- and 2-band (1&2-band) conditions (Figure 1). There was no significant difference (*p* > 0.05) in either accuracy, *d’*, or RT between 1 and 2 bands. By contrast, the 3-band condition yielded a significant improvement in all three performance measures compared to the 2-band condition (*p* = 0.000122, *p* = 0.000122, *p* = 0.00171, respectively). Performance at 3 and 4-bands was comparable in terms of accuracy, *d*’ and RT (*p* > 0.05 for all three measures). There was a significant improvement in performance from the 4-band to the clear condition (accuracy *p* = 0.000122, *d*’ *p* = 0.000122, RT *p* = 0.00403). Thirteen participants performed at near-ceiling level for clear speech (>90% accuracy, *d’* > 3). The remaining two had a hit rate of 81.3 and 83.8% (*d’* = 1.79 and 1.99, respectively), which nonetheless was an improvement over their performance in the 4-band condition.

The greatest variability in performance across participants occurred in the 3 and 4 band conditions. Average accuracy between the 3 and 4 band conditions was used as the general measure of participants’ task performance. With respect to this measure, participants were characterized as either good (78.8–91.3% accuracy), intermediate (66.3–70.6%) or poor (47.5–62.5%) performers, corresponding to three tertiles of the 3&4-band accuracy distribution. Importantly, 3&4-band accuracy and *d’* were highly correlated (*r* = 0.983, *p* < 0.0001). Based on the lack of significant differences in task performance between 1&2-band, and between 3&4-band conditions, iEEG data (see below) were pooled accordingly.

Participants’ demographic, audiometric, neuropsychological, and clinical background was examined to test whether any of these factors could account for performance variability (Supplementary Figure 3). No significant relationships were found between 3&4-band accuracy and demographic factors (sex, age), audiometry (PTA_0_._5–2_, SRT, WRS), neuropsychology (FSIQ, VCI, WMI) and participants’ clinical background (epilepsy age of onset, years of epilepsy, or localization of seizure focus to left or right hemisphere) (*p* > 0.05 for all comparisons). Participant R250, who had the best 3&4-band performance (91.3% accuracy), was also the oldest in the cohort (50 years old), had the worst hearing thresholds (PTA_0_._5–2_ 38.3 dB HL, SRT 30 dB HL), and the longest history of epilepsy (46 years; age of onset 4 years old). Additionally, participant L275, who had the lowest FSIQ, VIC and WMI scores, in part attributed to English being his second language by the neuropsychologist, exhibited above-average 3&4-band performance (70.6%).

### Examples of iEEG responses to spectrally degraded speech

**FIGURE 2:**
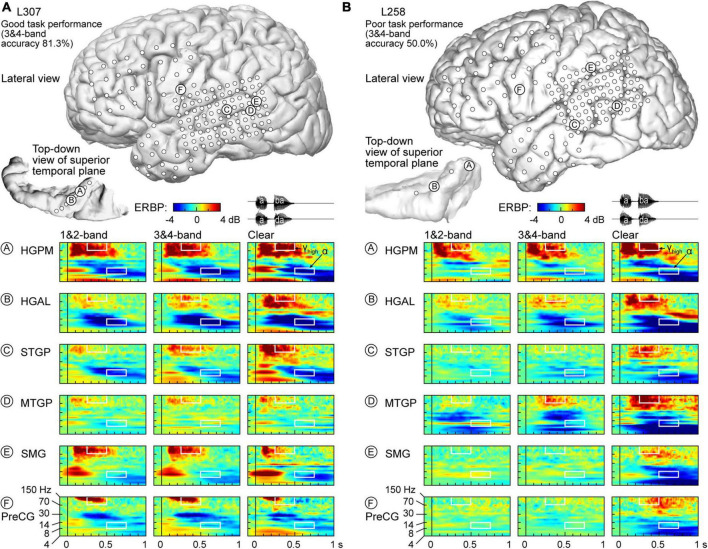
Responses to noise-vocoded and clear speech in two exemplar participants. **(A)** L307, who exhibited good task performance. Location of subdural arrays implanted over left hemispheric convexity and location of depth electrode contacts in the superior temporal plane are shown on top. Responses to noise-vocoded and clear sounds /aba/ and /ada/ (waveforms shown on top right) recorded from exemplar sites in the posteromedial and anterolateral portions of Heschl’s gyrus (HGPM, HGAL; sites A, B, respectively), posterior portion of the superior temporal gyrus (STGP; site C), middle portion of the middle temporal gyrus (MTGM; site D), supramarginal gyrus (site E), and precentral gyrus (PreCG, site F). ERBP is computed for iEEG frequencies between 4 and 150 Hz. Data (/aba/ and /ada/ trials combined) are averaged between 1- & 2- band, and between 3- & 4-band conditions. White rectangles denote time-frequency windows used to measure high gamma (250–500 ms, 70–150 Hz) and alpha (500–750 ms, 8–14 Hz) ERBP. **(B)** L258, who exhibited poor task performance.

**FIGURE 3:**
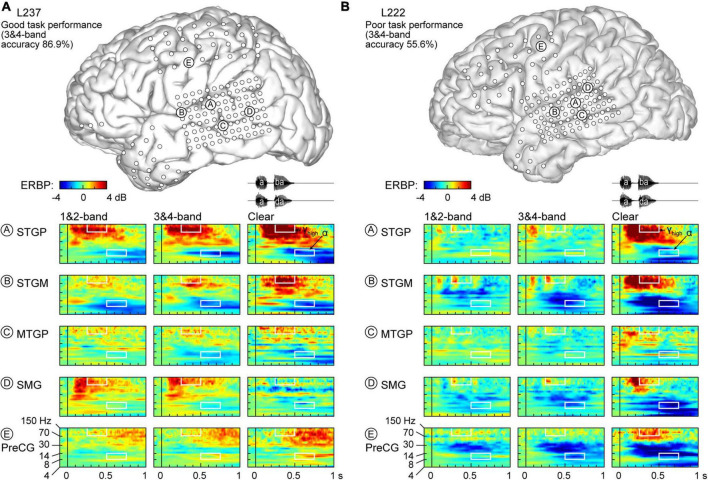
Responses to noise-vocoded and clear speech in two additional exemplar participants. **(A)** L237, who exhibited good task performance. **(B)** L222, who exhibited poor task performance. See caption of Figure 2 for details.

Physiology from four participants highlights the different patterns of cortical responses to the stimuli observed across cortical processing stages, stimulus conditions, and task performance (Figures 2, 3). Two of the four participants (L307, Figure 2A; L237, Figure 3A) performed the task with high accuracy in the 3&4-band condition, whereas the other two participants (L258, Figure 2B; L222, Figure 3B) had chance performance in the same condition. These participants were chosen as exemplars based on comparable electrode coverage within auditory cortex and other relevant cortical regions, with emphasis on the dorsal processing stream.

Figure 2A illustrates response patterns seen in a good performer whose 3&4-band accuracy was 81.3%. The top panel presents electrode coverage and the locations of exemplar recording sites A-F. Responses to the speech stimuli were characterized by increases in high frequency (>30 Hz) ERBP, with concomitant suppression of low frequency power. For clarity, high frequency ERBP augmentation will be described first. Activity within site A (HGPM) was characterized by strong increases in low and high gamma ERBP to both syllables. The strong high gamma augmentation was present regardless of spectral complexity or intelligibility of the stimuli. This pattern was operationally defined as *non-selective* (see Methods for formal definitions of specific response patterns). By contrast, site B (HGAL, 1 cm anterolateral to site A) showed a *clear-preferred* pattern, with comparable responses to the vocoded stimuli, and an elevated high gamma response to clear speech. At site C (STGP), there was a progressive increase in high gamma activity from 1&2-band to 3&4-band to clear speech (*spectral complexity pattern)*. At MTGP site D, the magnitude of responses was smaller than in the auditory cortex (cf. sites A-C), with comparable high gamma augmentation between adjacent conditions (non-selective response pattern). On the SMG and PreCG (site E and F), there were strong responses to the second syllable in the vocoded conditions, whereas activity was diminished in the clear condition (“*vocoded-preferred*” pattern).

Examples of physiology within the same ROIs for a representative poor performer (50.0% accuracy at 3&4-band) are shown in Figure 2B. Within HGPM site A and MTGP site D, during the second syllable there was a progressive increase in response to more *spectrally complex* stimuli, while site B within HGAL featured a *clear-preferred* pattern. Sites within STGP, SMG and PreCG (sites C, E, F) had an even stronger preference for clear speech, wherein vocoded stimuli failed to elicit high gamma activation altogether (*clear-specific* pattern).

Low frequency suppression was a prominent feature of responses to the stimuli in both participants presented in Figure 2. Within auditory cortex, suppression often began in the beta range, and extended into alpha and even theta bands. More variable profiles of low frequency suppression were observed outside the auditory cortex. While patterns of low frequency suppression often mirrored patterns seen in the high gamma frequency range, this was not always the case at the single participant level. This dissociation is exemplified in Figure 3, which presents data from two additional participants whose 3&4-band performance was 86.9% (Figure 3A) and 55.6% (Figure 3B). In the good performer L237, strong high gamma responses were not paralleled by similarly strong suppression of low frequency ERBP. In the poor performer L222, responses to vocoded speech were dominated by suppression with only transient high gamma responses to the two syllables. High gamma augmentation was primarily limited to the clear speech condition. The high gamma and alpha response patterns across other recordings sites in the four exemplar participants are provided in Supplementary Figure 4.

As observed in Figures 2, 3, low frequency suppression could occur to varying degrees in theta, alpha and beta bands. The relatively short ISIs (750–760 ms from the button press to the next trial onset) and the pre-stimulus time window used to estimate baseline power for ERBP calculation were too short for accurate quantitative analysis of theta ERBP. Changes in the beta band often preceded alpha suppression. Thus, it was important to determine whether these earlier ERBP changes in the beta band or the later reductions of power in the alpha band represent a more sensitive index of suppression. To that end, linear regression analysis was performed between the two metrics for the clear condition, with ERBP measured within 250–500 and 500–750 ms for beta and alpha, respectively (Supplementary Figure 5A). This analysis yielded the following relationship between alpha (*x*) and beta (*y*): *y* = 0.332*x-0.0104. The slope of < 1 indicates that alpha is a more sensitive metric of low frequency suppression. This was further examined by computing pairwise differences between beta and alpha ERBP across the auditory cortical hierarchy (Supplementary Figure 5B). The median difference between beta and alpha ERBP across all recording sites was 0.420, corresponding to a significantly greater degree of ERBP suppression when measured in the alpha band (*p* < 0.0001, Wilcoxon signed-rank test). This relationship was present in all examined ROI groups (*p* < 0.0001, Wilcoxon signed-rank tests) except prefrontal, where the beta-alpha difference failed to reach significance (median −0.0498 dB, *p* = 0.155). Thus, quantitative analysis of low frequency suppression focused on the alpha band, where ERBP was measured within the time window of 500–750 ms after stimulus onset.

### High gamma augmentation and alpha suppression patterns in the left and the right hemisphere

**FIGURE 4:**
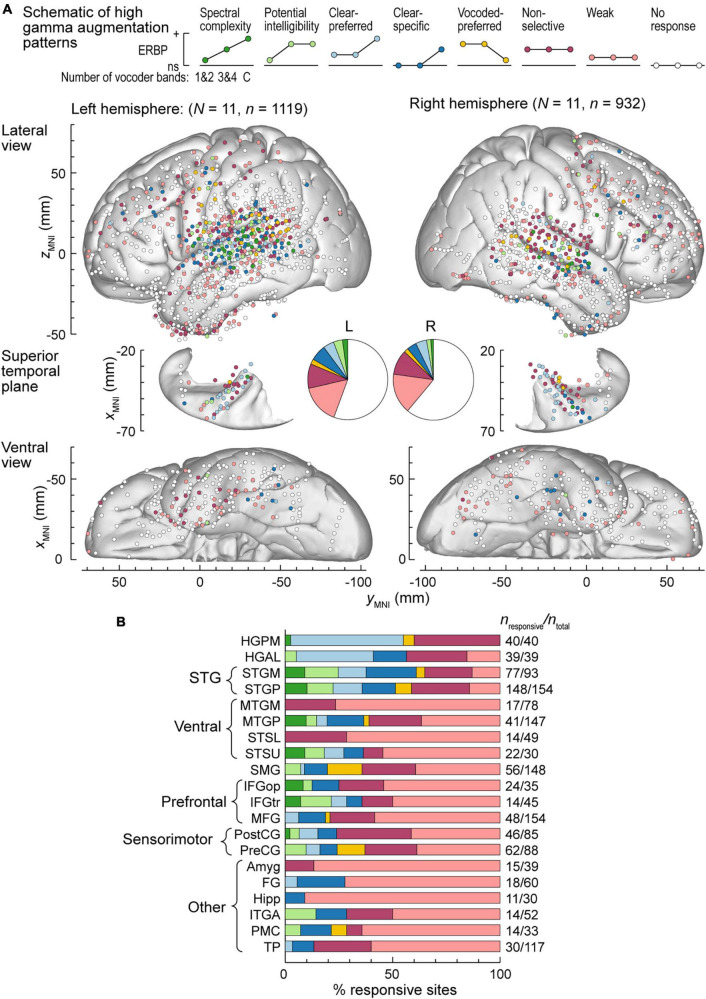
High gamma augmentation patterns in the left and right hemispheres. **(A)** Summary of data from all participants plotted in the MNI coordinate space and projected onto the FreeSurfer average template brain for spatial reference. Side views of the lateral hemispheric convexity, top-down views of the superior temporal plane and ventral views are aligned along the *y*_MNI_ axis. Schematics of the eight high gamma augmentation patterns are presented on top. **(B)** Distribution of high gamma augmentation patterns throughout the auditory cortical hierarchy. Each horizontal bar depicts percentages of sites characterized by different high gamma augmentation patterns in a ROI as a fraction of the number of responsive sites in that ROI (i.e., excluding the “No response” pattern). Numbers of responsive sites and total numbers of sites in each ROI with > 10 responsive sites from at least two participants are shown on the right. See schematic in panel a for color coding of the patterns.

Exemplar data from Figures 2, 3 were from participants with electrodes overlying the left hemisphere. Comparable coverage of left and right hemispheres in the participant cohort (1,119 sites from 11 participants and 932 sites from 11 participants, respectively; see Methods) permitted examination of hemispheric differences and regional distribution in response patterns. Figure 4A illustrates electrode locations in all participants based on MNI coordinates, as shown in lateral, superior temporal plane and ventral views of the brain. Individual sites are color-coded according to the pattern of gamma augmentation (see schematics on top of Figure 4A). Pie charts in the center of Figure 4A summarize the patterns of responses seen in both hemispheres at the whole-brain level. There was comparable activation in both hemispheres, with similar distributions of response types. At the level of individual ROIs, none were found to exhibit significant left-right differences in terms of pattern distribution (*p* > 0.05, Fisher exact tests, FDR-corrected).

Figure 4B summarizes regional distribution of response patterns for ROIs with > 10 responsive sites (data for all ROIs are presented in Supplementary Table 1). As expected, canonical auditory cortex (HGPM, STP, STG) had the highest prevalence of responsive sites, with non-selective and clear-preferred responses particularly prominent in HGPM and HGAL. Responses reflecting spectral complexity or potential intelligibility of the stimuli were typically found within STG, adjacent auditory-related ROIs (MTGP, STSU) and, to a more limited degree, within ventrolateral prefrontal ROIs IFGop and IFGtr. Overall, responses outside canonical auditory cortex were characterized by a large percentage of sites with only weak activation. Notable exceptions included SMG and PreCG, two regions within the dorsal auditory cortical pathway. Of note, a total of 39 sites exhibited vocoded-preferred responses. Of these, the majority were localized to STGP (*n* = 11), SMG (*n* = 9) and PreCG (*n* = 8), 3 were in STGM, and the remaining 8 were scattered in other brain regions.

**FIGURE 5:**
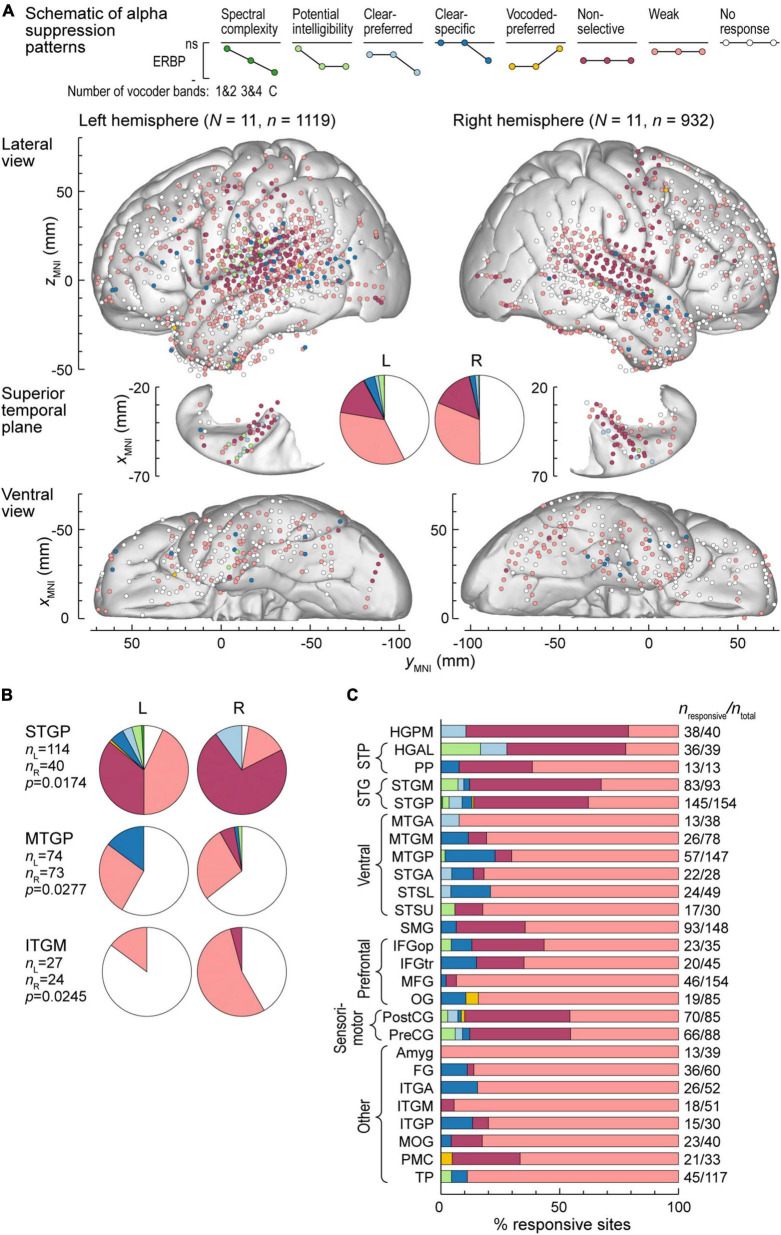
Alpha suppression patterns in the left and right hemispheres. **(A)** Summary of data from all participants plotted in the MNI coordinate space and projected onto the FreeSurfer average template brain for spatial reference. See caption of Figure 4A for details. **(B)** Pie charts depicting proportions of different alpha suppression patterns in ROIs that exhibited significant (*p* < 0.05, Fisher exact tests, FDR-corrected) differences between the left and the right hemisphere. See schematic in panel **(A)** for color coding of the patterns. **(C)** Distribution of alpha suppression patterns throughout the auditory cortical hierarchy. See caption of Figure 4B for details.

Alpha suppression was predominantly non-selective or weak, with similar overall distribution between the two hemispheres (Figure 5A). Three ROIs exhibited significant hemispheric differences, as assessed by Fisher exact tests. These were STGP, MTGP and ITGM, with a higher prevalence of non-selective alpha suppression in the right STGP, a higher prevalence of clear-specific pattern in the left MTGP and a higher prevalence of weak responses in the right ITGM (Figure 5B). Figure 5C summarizes regional distribution of alpha suppression patterns for ROIs with > 10 responsive sites (data for all ROIs are presented in Supplementary Table 2). Non-selective alpha suppression was most common within canonical auditory cortex, while higher-order ROIs were more typically characterized by the weak suppression profile. In contrast to high gamma augmentation, alpha suppression patterns that reflected sensitivity to stimulus condition (spectral complexity, potential intelligibility, clear- or vocoded preferred and clear-specific) were much less common (cf. Figure 4B), suggesting that modulation of alpha power *per se* does not encode these acoustic attributes of the stimuli.

The relatively high prevalence of iEEG responses to vocoded stimuli measured in the PreCG raises the possibility that these responses could be related to motor activity rather than auditory sensory processing. This possibility was addressed in three ways. First, participants had to press a button following each trial, using their left hand for an /aba/ and their right hand for an /ada/ response. Thus, motor-related iEEG activity in the left PreCG would be expected to be greater when associated with /ada/ responses (i.e., contralateral hand) than /aba/, and vice versa for the right PreCG. None of the 83 PreCG sites exhibited a significant (*p* < 0.05) difference between high gamma augmentation or alpha suppression associated with /aba/ vs, /ada/ behavioral responses. Second, analysis windows for high gamma and alpha ERBP (250–500 and 500–750 ms, respectively) preceded button press times. Specifically, 500 and 750 ms corresponded to 0.227th and 4.61st percentile, respectively, of the RT distribution in the 11 participants who had PreCG coverage. Finally, the distribution of responsive sites was examined with respect to motor somatotopy of the PreCG (Roux et al., 2020). This comparison revealed that electrode coverage of the PreCG was spatially distinct from wrist and fingers areas that would be expected to be involved in the motor response (Supplementary Figure 6). Instead, the extent of PreCG coverage and the locations of sites with significant high gamma or alpha responses to speech broadly overlapped with lips, tongue, and larynx regions of PreCG. Taken together, these findings argue against the interpretation of recorded activity as being associated solely with the motor response or the preparatory activity preceding the motor action. Instead, the likely interpretation is that high gamma augmentation and alpha suppression recorded in the PreCG are sensory in nature. These findings are consistent with recent literature highlighting contributions of PreCG to speech perception (Wong et al., 2008; Murakami et al., 2012; Cheung et al., 2016).

### High gamma augmentation and alpha suppression patterns in good and poor performers

**FIGURE 6:**
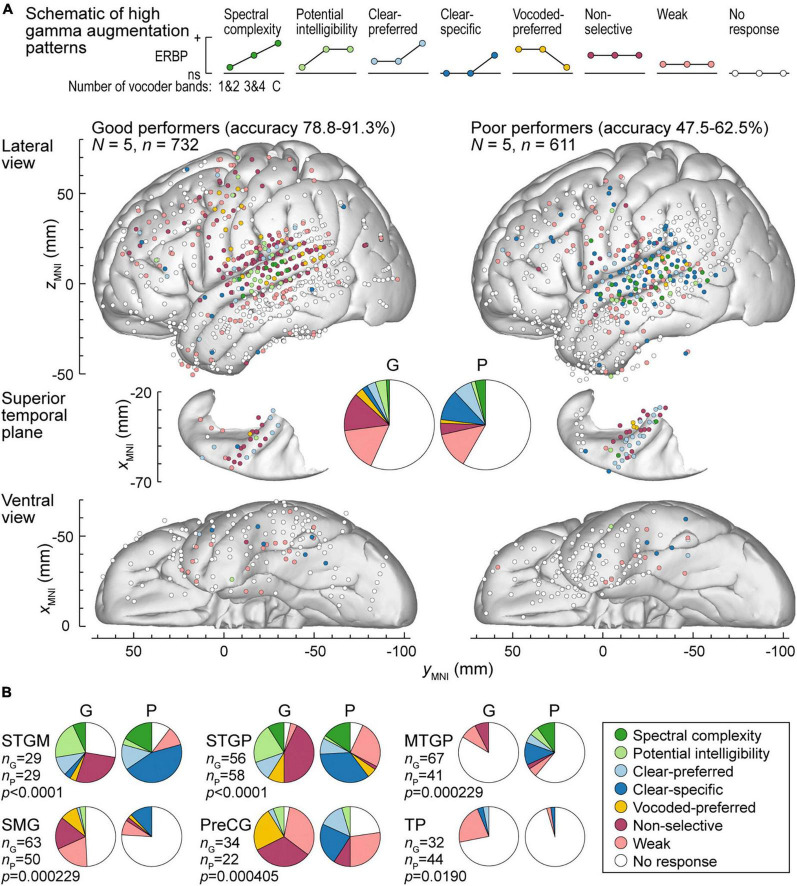
High gamma augmentation patterns in participants who exhibited good and poor performance in the behavioral task (left and right panels, respectively). **(A)** Summary of data plotted in MNI coordinate space and projected onto the left hemisphere of the FreeSurfer average template brain for spatial reference. Right hemisphere MNI *x*-axis coordinates (*x*_MNI_) were multiplied by –1 to map them onto the left-hemisphere common space. Side views of the lateral hemispheric convexity, top-down views of the superior temporal plane and ventral views are aligned along the *y*_MNI_ axis. Schematics of the eight high gamma augmentation patterns are presented on top. **(B)** Pie charts depicting proportions of different high gamma augmentation patterns in ROIs that exhibited significant (*p* < 0.05, Fisher exact tests, FDR-corrected) differences between good and poor performing participants.

**FIGURE 7:**
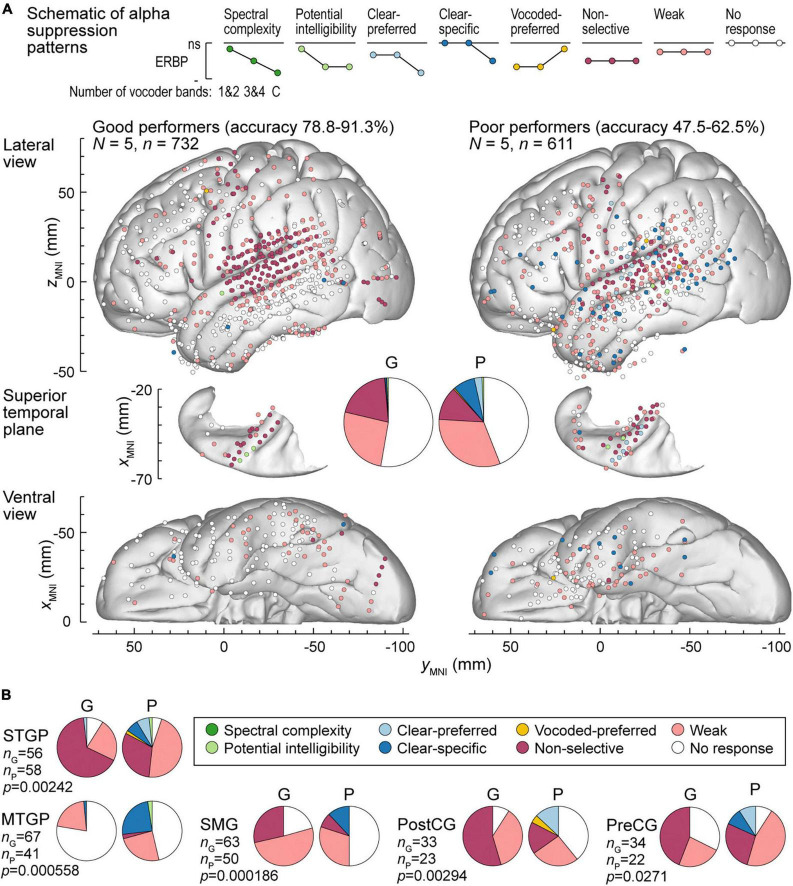
Alpha suppression patterns in participants who exhibited good and poor performance in the behavioral task (left and right panels, respectively). **(A)** Summary of data plotted in MNI coordinate space and projected onto the left hemisphere of the FreeSurfer average template brain for spatial reference. **(B)** Pie charts depicting proportions of different alpha augmentation patterns in ROIs that exhibited significant (*p* < 0.05, Fisher exact tests, FDR-corrected) differences between good and poor performing participants. See caption of Figure 6 for details.

Patterns of high gamma and alpha ERBP were examined with respect to participants’ task performance. Focus was placed on the five participants who performed best in the 3&4-band condition, and the five participants who performed the worst in the same condition (Figures 6, 7). Figure 6A illustrates the distribution of high gamma response types in these two groups. On the whole brain level, the greatest difference was the larger percentage of sites showing non-selective high gamma augmentation in good performers (good: 13.8%, poor: 4.09%) contrasting with the larger percentage of clear-specific responses in the poor performers (good: 2.05%, poor: 11.1%) (pie charts in Figure 6A). This reflects weaker cortical activation by spectrally degraded speech (1&2 and 3&4 band conditions) in poor performers.

The differences between good and poor performers were not uniform across ROIs. Along Heschl’s gyrus, responses were predominantly non-selective or clear-preferred in both groups of participants. Despite the overall low prevalence of the vocoded-preferred pattern (good: 3.01%, poor: 1.31%), it was predominantly seen in good performers along the STG and extending into the SMG and PreCG. Within prefrontal cortex, the three ROIs with sufficient electrode coverage to permit quantitative comparisons (IFGtr, MFG and OG) did not exhibit systematic differences between the two groups. Overall, prefrontal cortex was characterized by a relatively low prevalence of responses, with weak activation being the predominant pattern.

Figure 6B summarizes those ROIs where there were significant differences between the two behavioral groups, as determined by Fisher exact tests with FDR correction for multiple comparisons. Significant differences were noted in both STGM and STGP. In these ROIs, good performers had a greater degree of non-selective high gamma augmentation, whereas responses in the poor group were predominantly clear-specific. MTGP showed significant differences between the two groups. In poor performers, MTPG appeared to be more strongly engaged in the processing of the stimuli, with diverse patterns reminiscent of those seen in STGM and STGP in good performers. Similarly, a major difference between good and poor performers was seen in SMG and PreCG, where poor task performance was associated with higher prevalence of clear speech-specific high gamma responses. Further, SMG in poor performers appeared to be less engaged in processing of the stimuli, with fewer responsive sites mainly represented by clear-specific or weak pattern.

Alpha suppression was also different between good and poor performers (Figure 7A). At the whole brain level, clear-specific alpha suppression was more prevalent in the poor performers. Significant differences at the ROI level were observed in STGP, MTGP, SMG, PostCG and PreCG (Figure 7B). In MTGP and SMG, the principal difference was a higher prevalence of clear-specific responses in poor performers. In the MTGP, there were either weak or no responses in good performers, suggesting that the task poorly activated this region in this behavioral group. In the STGP, PreCG and PostGG the dominant pattern in good performers was non-selective suppression, contrasting with poor performers where clear-preferred or clear-specific profiles were present. Taken together, good performance was associated with non-selective alpha suppression patterns, where even the 1&2-band condition elicited significant cortical responses, whereas poor performers were characterized by a higher prevalence of clear-specific responses.

To further test the reliability of response differences between good and poor performers, the distribution of high gamma and alpha response patterns was also examined in the 5 participants who exhibited intermediate performance (66.3–70.6% hit rate; see Figure 1). In this group, the percentages of sites with non-selective and clear-specific high gamma activation (15.2 and 5.34%, respectively) were intermediate relative to the good (16.0 and 1.95%) and the poor (2.61 and 15.4%) performing groups (Supplementary Figure 7A). Likewise, percentages of sites with non-selective and clear-specific alpha suppression patterns were intermediate in this group (Supplementary Figure 7B). In summary, this comparison suggests a graded relationship between cortical response patterns and task performance across the participant cohort studied here.

The qualitative assessment of high gamma and alpha response patterns presented above was supplemented by quantitative analyses to examine the relationship between participants’ cortical responses to vocoded and clear stimuli and task performance, characterized by the average accuracy in the 3&4-band condition. The results of Spearman rank correlation analysis for STGP, MTGP, SMG, PreCG, PostCG, and TP are presented in Supplementary Figure 8. These six ROIs exhibited significant pattern distribution differences between good and poor performers (see Figures 6B, 7B). In STGM, Spearman’s rank correlations did not reach significance in any of the six comparisons (*p* < 0.05, FDR-corrected; data not shown).

In STGP, SMG and PreCG, there was a significant positive correlation between high gamma ERBP in response to vocoded (but not clear) speech and task performance (STGP: *ρ* = 0.591, *p* < 0.0001 for 1&2-band, *ρ* = 0.557, *p* < 0.0001 for 3&4-band; *ρ* = 0.166, *p* = 0.128 for clear speech; SMG: *ρ* = 0.294, *p* = 0.00217 for 1&2-band, *ρ* = 0.267, *p* = 0.00701 for 3&4-band; *ρ* = 0.0844, *p* = 0.511 for clear speech; PreCG: *ρ* = 0.420, *p* = 0.000614 for 1&2-band, *ρ* = 0.455, *p* = 0.000151 for 3&4-band; *ρ* = 0.199, *p* = 0.168 for clear speech). The SMG was characterized by significant negative correlations between alpha ERBP in response to vocoded (but not clear) speech and average accuracy in the 3&4-band condition (*ρ* = −0.272, *p* = 0.0174 for 1&2-band, *ρ* = – 0.473, *p* < 0.0001 for 3&4-band; *ρ* = 0.0539, *p* = 0.787 for clear speech). That is, better performance was associated with greater alpha suppression in response to all vocoded stimuli. Additionally, there was a significant correlation between alpha suppression in the 1&2-band condition and 3&4-band performance in the STGP (*ρ* = – 0.301, *p* = 0.00399), and between alpha suppression in the 3&4-band condition and 3&4-band performance in the PostCG (*ρ* = – 0.352, *p* = 0.0174).

A different relationship between alpha suppression and task performance was found in MTGP. Here, greater alpha suppression in response to 3&4-band and clear stimuli was associate with worse task performance (*ρ* = 0.263, *p* = 0.0195 for 3&4-band; *ρ* = 0.333, *p* = 0.00200 for clear speech). Thus, there was a fundamental difference in processing of speech in MTGP between good and poor performers, inverse to that seen in other ROIs. Finally, there was a significant correlation between high gamma responses to clear speech and 3&4-band performance in the TP (*ρ* = 0.352, *p* = 0.00105). However, as responses in the TP were generally weak or absent, the significance of this finding is uncertain. In summary, there is general agreement between the qualitative and quantitative analyses, and results emphasize the importance of the dorsal auditory cortical stream in discrimination of stop consonants with degraded spectral information.

## Discussion

### Summary of findings

In the current study, we leveraged intracranial recordings from more than 2,000 contacts with high spatiotemporal fidelity to examine cortical responses to vocoded speech of varying degradation. Several key results were obtained that advance our understanding of variability in perception of degraded speech: (1) Participants showed variability in performance when listening to 3&4-band vocoded speech, suggesting that this is a useful proxy for understanding speech perception in CI users. (2) Better task performance was associated with greater recruitment of regions along the dorsal auditory processing stream (STGP, SMG, PreCG) in response to vocoded stimuli. (3) In contrast, poor performers exhibited a greater involvement of the ventral stream (MTGP) when listening to clear speech. These results suggest differences in the balance of neural processing along the dorsal and ventral stream between good and poor performers.

### Variability in task performance

For spectrally degraded speech to serve as a model for the variability in CI outcomes, its associated behavioral and physiologic characteristics should be comparable to those in CI users. This was accomplished with the 3&4-band condition, where variability in perception across hearing participants allowed for the identification of three groups, performing at good, intermediate, and poor levels. Poor performers exhibited ceiling or near-ceiling performance in the clear condition, indicating that deficiency seen with spectrally degraded speech was not based on more general factors such as degree of attention. The overall performance profiles seen in the present cohort are consistent with those reported previously in studies of spectrally degraded speech stimuli in hearing individuals (Shannon et al., 1995; Scott et al., 2006). The stratification based on place of articulation is particularly relevant given that information transmission associated with this attribute exhibits the greatest difference between overall better and poorer CI listeners compared to voicing, manner, or duration (Munson et al., 2003).

A notable finding was the lack of relationships between task performance and peripheral hearing. This suggests that variability in performance can be attributed to individual differences at the level of the central nervous system rather than the auditory periphery. The current study was able to identify manifestations of this variability at the cortical level. Future studies that utilize non-invasive recordings in human participants (Anderson et al., 2020; Hernández-Pérez et al., 2021) and direct recordings in animal models (Ranasinghe et al., 2013) may shed light on the subcortical contributions to this variability.

### Responses to noise-vocoded and clear speech in canonical auditory cortex

Within core auditory cortex, high gamma augmentation was either non-selective (significant responses to all stimuli, comparable in magnitude across conditions) or followed the clear-preferred pattern (with a significantly larger responses to clear speech compared to the 3&4-band condition). One factor that can contribute to the clear-preferred response profile is phase locking to the fundamental frequency of the male talker in the clear condition (Steinschneider et al., 2013; Simon et al., 2022). This contribution would not be present in response to noise-vocoded stimuli where the spectral energy peak associated with the fundamental frequency would be smeared within the corresponding vocoder frequency band. As the fundamental frequency was within the high gamma iEEG frequency range, frequency-following responses at the level of HGPM could contribute to the measured high gamma ERBP. To eliminate this contribution, it would be necessary to use utterances by speakers with fundamental frequencies above the limit of phase locking in HGPM (e.g., most female speakers; Hillenbrand et al., 1995).

Phase-locking to the speakers’ fundamental frequency is less likely to contribute to measured high gamma activity in non-core auditory cortical ROIs (Nourski et al., 2013a). These areas featured a greater diversity in response patterns, including the emergence of responses reflecting spectral complexity and, at the extreme, responding only to clear speech. This transformation is consistent with neuroimaging studies that demonstrated increased preference for complex stimuli along the auditory cortical hierarchy (Wessinger et al., 2001; Scott et al., 2006; Chevillet et al., 2011). While not a common pattern, multiple sites within STGP responded more strongly to vocoded than clear stimuli. This response pattern may reflect recruitment of additional resources for perception of spectrally degraded speech, corresponding to increased effort and greater task difficulty (Peelle, 2018).

Non-selective responses in STGM and STGP were common in good performers compared to a higher prevalence of clear-specific pattern in poor performers. This finding suggests that in poor performers, noise-vocoded stimuli are “rejected” at the level of non-core auditory cortex as non-speech sounds. In contrast, non-core auditory cortex in good performers processes even severely degraded stimuli, treating these sounds as potentially intelligible speech. This interpretation is supported by studies showing that electrical stimulation or strokes of these regions disrupts speech perception (Boatman and Miglioretti, 2005; Hamilton et al., 2021; Rogalsky et al., 2022). Functionally, high gamma activity in the STG is related to the transformation from acoustic to phonemic encoding (Mesgarani et al., 2014; Hamilton et al., 2021). It follows that the functions of STGM and STGP are key components in the decoding of spectrally degraded speech.

### Responses to noise-vocoded and clear speech in auditory-related cortex

Beyond canonical auditory cortex, there was a general decrease in activation to the syllables, with several notable exceptions. These include STSU, MTGP, SMG, PreCG, and PostCG. Each of these areas, with the exception of MTGP, is involved in phonologic processing. MTGP both in normal hearing listeners and CI users is considered a region important for lexico-semantic processing (Hickok, 2009; Song et al., 2015; Youssofzadeh et al., 2022). STSU can be considered part of Wernicke’s area with special emphasis on prelexical speech processing (Okada et al., 2010; Liebenthal et al., 2014). The other three ROIs are components of the dorsal auditory pathway whose key function is envisioned to be audiomotor integration (Hickok and Poeppel, 2007; Chang et al., 2015; Rauschecker, 2018).

Multiple observations support the importance of the dorsal auditory cortical stream in the decoding of spectrally degraded speech. Vocoded-preferred high gamma responses were most commonly observed within STGP, SMG, and PreCG. This apparent recruitment of sites along the dorsal stream to process vocoded speech contrasts with other studies that have identified regions in prefrontal cortex that are recruited under challenging listening conditions (Rovetti et al., 2019; White and Langdon, 2021; Zhou et al., 2022). This discrepancy is likely based upon task differences, wherein the task in the other studies was sentence-level recognition and semantic processing as opposed to the phonemic task examined here.

High gamma response patterns within STGP, SMG, and PreCG differed between good and poor performers. Non-selective responses were prominent in good performers whereas clear-specific responses were the dominant response patterns in poor performers. Multiple studies have shown that SMG plays a role in phonemic processing (Turkeltaub and Coslett, 2010; Zevin et al., 2010). Activation within SMG by vocoded speech in naïve listeners has also been associated with subsequent improved performance following training (Lin et al., 2022). There is growing evidence for the importance of PreCG and PostCG in speech perception at the phonemic level (Cogan et al., 2014; Cheung et al., 2016) through the process of using articulatory cues (Pulvermüller et al., 2006; D’Ausilio et al., 2009; Schomers et al., 2015) or mapping of acoustic speech attributes onto their articulatory representations (Cheung et al., 2016).

Similar response pattern differences distinguishing good from poor performers were also observed for alpha suppression. As a general rule, there was a greater prevalence of non-specific responses in good performers and more common clear-specific and clear-preferred responses in poor performers. Once again, these differences were present in ROIs along the auditory cortical dorsal pathway. Thus, both metrics emphasize the importance of the dorsal auditory stream in processing the phonemic attributes of vocoded speech. These observations have translational relevance for non-invasive studies using EEG and MEG, where inferences can be made from the robust lower frequency changes with regard to likely changes in the low amplitude signals afforded by high gamma activity.

It is interesting to speculate upon the differences between good and poor performers in MTGP. For high gamma activity, poor performers demonstrated a higher overall response prevalence with a greater diversity of response patterns. Alpha suppression was minimal in good performers, whereas a significant degree of clear-specific responses were seen in poor performers. These observations suggest the intriguing idea that poor performers engage the ventral pathway to a greater extent than the good performers in this phonemic identification task. This interpretation is supported by the findings of Rogalsky et al. (2022), where deficits in comprehension but not discrimination tasks were noted for strokes within MTGP, whereas deficits in phonemic discrimination were noted in more dorsal sensorimotor regions.

### Caveats

As is the case for all iEEG studies, the question can be raised whether results obtained in a cohort of neurosurgical patients are representative of the general population. Examination of the demographic, audiometric, neuropsychological and neurological backgrounds (see Supplementary Figure 3) failed to identify clinical factors that might affect task performance in the 3&4-band condition. Further, all participants were able to successfully perform the task at near-ceiling levels with clear speech. With regard to the patients’ clinical background, epileptic foci were excluded from the analyses, and participants were not tested after a seizure unless they were alert and back to neuropsychological baseline.

A related question is the extent to which results obtained in hearing individuals can be extrapolated to the CI user population. While noise vocoding is a commonly used approach to model the input to the central auditory system in CI listeners (e.g., Rosen et al., 1999; Başkent and Shannon, 2004), there is no ideal way to exactly match what a CI user perceives (Karoui et al., 2019; Dorman et al., 2020). Future work examining the perceptual experience in CI users with single-sided deafness will be helpful in addressing this important question.

Another caveat is that results of speech and language testing are strongly biased by the specific tasks used (Hickok and Poeppel, 2007). The current 2AFC task was based upon place-of-articulation phonemic identity of the consonant. A different task design (e.g., four-interval 2AFC or open-set identification) with the same type of stimuli might lead to different behavioral outcomes across the studied participant cohort (Gerrits and Schouten, 2004). Given the very high congruence between accuracy and sensitivity (*d’*) in the present study, it is likely that the overall breakdown of the cohort into good, intermediate and poor performers would be stable had a different place of articulation task been used.

Current findings emphasized the importance of the dorsal auditory processing stream for the successful completion of this task. A different task that requires semantic processing such as identifying words that belong to a specific category or sentence-level comprehension of spectrally degraded speech may strongly engage the ventral stream and recruit prefrontal cortex to a greater degree than seen in the present study (Peelle, 2018). Additional studies using more complex experimental designs will be required to test this hypothesis.

Despite the extensive iEEG coverage of multiple brain areas, it must be acknowledged that there were several relevant ROIs that were not adequately sampled. Specifically, there was limited sampling of PT, PP, and InsP – regions immediately adjacent to HGPM and HGAL. These areas are key components of the cortical auditory networks involved in speech processing (Zhang et al., 2019; Hamilton et al., 2021; Nourski et al., 2022). Likewise, STSU, another area important for phonemic processing, had limited coverage. Consequently, absence of findings in these ROIs should not be construed as evidence that these regions do not contribute to the processing of degraded speech.

### Future directions

In addition to studies directed at semantic processing of vocoded speech, future work must also address cortical underpinnings of training and experience. The degree of neural recruitment can be predicted to decrease with improvements in task performance over time. This issue is particularly relevant to CI users where recruitment of neural resources for sensory processing may occur to the detriment of these resources being available for cognitive processing (Pichora-Fuller et al., 1995; Gao et al., 2023). From the clinical standpoint, future studies may aid in the development of novel objective measures to assess CI performance and effects of training based on activation patterns within key brain areas such as STGP and SMG. These measures would be of special import in pediatric populations where accurate behavioral report-based testing may not be feasible. Finally, neuromodulation-based rehabilitation strategies are gaining momentum in clinical practice, as exemplified by the use of neurofeedback in tinnitus patients based on alpha power (Hartmann et al., 2014). While implanted hardware makes conventional neuromodulation strategies challenging, new generation CI devices may permit transcranial magnetic stimulation which has not been considered feasible in CI users until recently (Mandalà et al., 2021). The present study suggests that cortical areas along the dorsal processing stream can be potential targets for such interventions.

## Data availability statement

The raw data supporting the conclusions of this article will be made available by the authors upon request, and upon establishment of a formal data sharing agreement.

## Ethics statement

The studies involving humans were approved by the University of Iowa Institutional Review Board. The studies were conducted in accordance with the local legislation and institutional requirements. The participants provided their written informed consent to participate in this study.

## Author contributions

KN: Conceptualization, Data curation, Formal analysis, Investigation, Methodology, Project administration, Software, Supervision, Validation, Visualization, Writing—original draft, Writing—review and editing. MS: Conceptualization, Methodology, Writing—original draft, Writing—review and editing. AR: Conceptualization, Investigation, Methodology, Writing—review and editing. JB: Formal analysis, Writing—review and editing. ED: Data curation, Formal analysis, Writing—review and editing. HK: Investigation, Methodology, Resources, Supervision, Writing—review and editing. MH: Funding acquisition, Resources, Supervision, Writing—review and editing.

## Footnotes

1
https://users.sussex.ac.uk/~cjd/

2
https://github.com/ckovach/DBT

3
https://www.R-project.org/
