# Supplementary material for: Intracranial electrophysiology of spectrally degraded speech in the human cortex
Source: Front Hum Neurosci. 2024 Jan 22;17:1334742. doi: 10.3389/fnhum.2023.1334742 (PMC10839784; doi:10.3389/fnhum.2023.1334742)
Supplement: Supplementary file 1 [file Data_Sheet_1.pdf]

Supplementary Material

Intracranial electrophysiology of spectrally degraded speech in the human cortex

Kirill V. Nourski*, Mitchell Steinschneider*, Ariane E. Rhone, Joel I. Berger, Emily R. Dappen, Hiroto Kawasaki, Matthew A. Howard III

*** Correspondence:** Corresponding Author: kirill-nourski@uiowa.edu

1 Supplementary Figures and Tables

1.1 Supplementary Figures

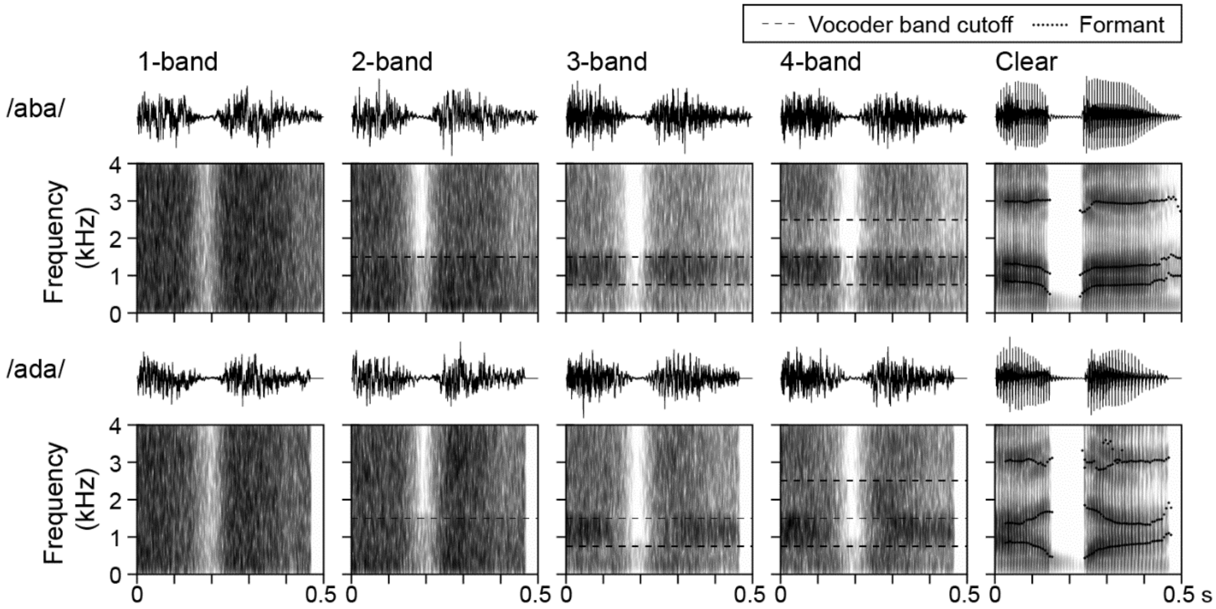

Supplementary Figure 1. Experimental stimuli /aba/ and /ada/ used in the task. Stimulus waveforms and spectrograms are shown in the top and bottom graphs, respectively. For vocoded stimuli, vocoder band cutoff frequencies are plotted over the spectrograms as dashed lines. For clear stimuli, the first three formants, estimated in Praat v.5.2.03, are plotted over the spectrograms as dotted lines.

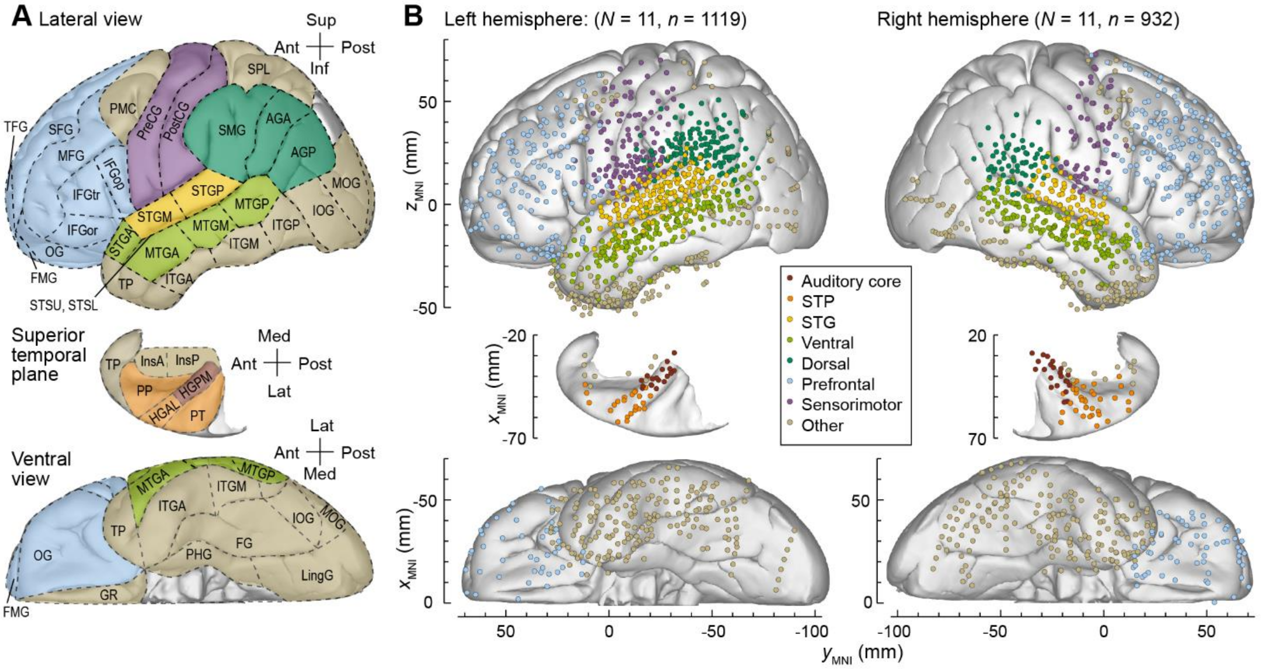

C

ROI group	Abbreviation	ROI	*N*	*n*
Auditory core	HGPM	Heschl's gyrus, posteromedial portion	11	40
STP	HGAL	Heschl's gyrus, anterolateral portion	11	39
	PP	Planum polare	3	13
	PT	Planum temporale	3	6
STG	STGM	Superior temporal gyrus, middle portion	10	93
	STGP	Superior temporal gyrus, posterior portion	11	154
Ventral	MTGA	Middle temporal gyrus, anterior portion	10	38
	MTGM	Middle temporal gyrus, middle portion	11	78
	MTGP	Middle temporal gyrus, posterior portion	12	147
	STGA	Superior temporal gyrus, anterior portion	8	28
	STSL	Superior temporal sulcus, lower bank	6	49
	STSU	Superior temporal sulcus, upper bank	9	30
Dorsal	AGA	Angular gyrus, anterior portion	7	31
	AGP	Angular gyrus, posterior portion	3	12
	SMG	Supramarginal gyrus	10	148
Prefrontal	ACC	Anterior cingulate cortex	1	5
	FMG	Frontomarginal gyrus	1	1
	IFGop	Inferior frontal gyrus, pars opercularis	12	35
	IFGor	Inferior frontal gyrus, pars orbitalis	2	2
	IFGtr	Inferior frontal gyrus, pars triangularis	10	45
	MFG	Middle frontal gyrus	12	154
	OG	Orbital gyri	11	85
	SFG	Superior frontal gyrus	5	31
	TFG	Transverse frontopolar gyrus	6	22

ROI group	Abbreviation	ROI	*N*	*n*
Sensori-motor	PostCG	Postcentral gyrus	11	85
	PreCG	Precentral gyrus	11	88
Other	Amyg*	Amygdala	13	39
	CingMA*	Cingulate gyrus, middle-anterior portion	1	1
	CingPD*	Cingulate gyrus, posterior-dorsal portion	1	3
	Cun*	Cuneus	2	11
	FG	Fusiform gyrus	10	60
	fOperc*	Frontal operculum	2	5
	GR	Gyrus rectus	5	13
	Hipp*	Hippocampus	11	30
	InsA	Anterior insula	4	10
	InsP	Posterior insula	5	10
	IOG	Inferior occipital gyrus	2	13
	ITGA	Inferior temporal gyrus, anterior portion	10	52
	ITGM	Inferior temporal gyrus, middle portion	8	51
	ITGP	Inferior temporal gyrus, posterior portion	7	30
	LingG*	Lingual gyrus	3	17
	MOG	Middle occipital gyrus	5	40
	OP	Occipital pole	1	5
	PHG	Parahippocampal gyrus	8	31
	PMC	Premotor cortex	11	33
	pOperc*	Parietal operculum	1	1
	Precun	Precuneus	1	5
	SOG	Superior occipital gyrus	1	6
	SPL	Superior parietal lobule	2	9
	TP	Temporal pole	11	117

*not pictured

Supplementary Figure 2. ROIs and electrode coverage in all 15 participants. **(A)** ROI parcellation scheme. **(B)** Locations of recording sites, determined for each participant individually and color-coded according to the ROI group, are plotted in MNI coordinate space and projected onto the FreeSurfer average template brain for spatial reference. Projections are shown in the lateral, top-down (superior temporal plane), and ventral views. Sites in the amygdala (*n* = 39), middle-anterior portion of the cingulate gyrus (*n* = 1), postero-dorsal portion of the cingulate gyrus (*n* = 3), cuneus (*n* = 4), frontal operculum (*n* = 5), hippocampus (*n* = 30), lingual gyrus (*n*=17), and parietal operculum (*n* = 1) are not shown. **(C)** ROI groups, ROIs and abbreviations used in the study. Number of participants and number of sites that contributed to each ROI are provided in the two right-most columns (*N* and *n*, respectively).

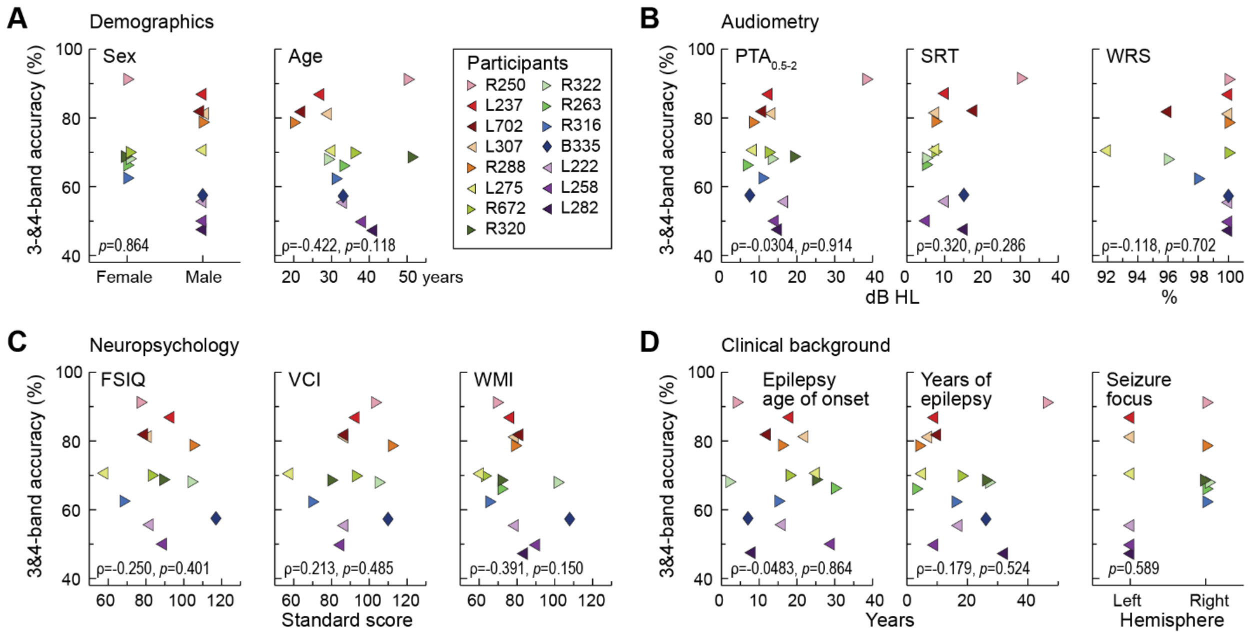

Supplementary Figure 3. Relationship between participants' demographic **(A)**, audiometric **(B)**, neuropsychological **(C)**, and clinical **(D)** background, relative to task performance (3&4-band accuracy). Left- and right-pointing arrowhead symbols represent participants with mainly left- and right hemisphere electrode coverage, respectively. Participant B335, with bilateral electrode coverage, is denoted by a diamond. Comparisons between female and male participants, and between participants with left- and right hemisphere seizure foci were done using Wilcoxon rank-sum tests; all other analyses were performed using Spearman's rank correlation. $PTA_{0.5\text{-}2}$, pure tone average (500, 1000, 2000 Hz; left-right ear average); SRT, speech reception threshold (left-right ear average); WRS, word recognition score (left-right ear average); FSIQ, Full-scale intelligence quotient; VCI, verbal comprehension index; WMI, working memory index.

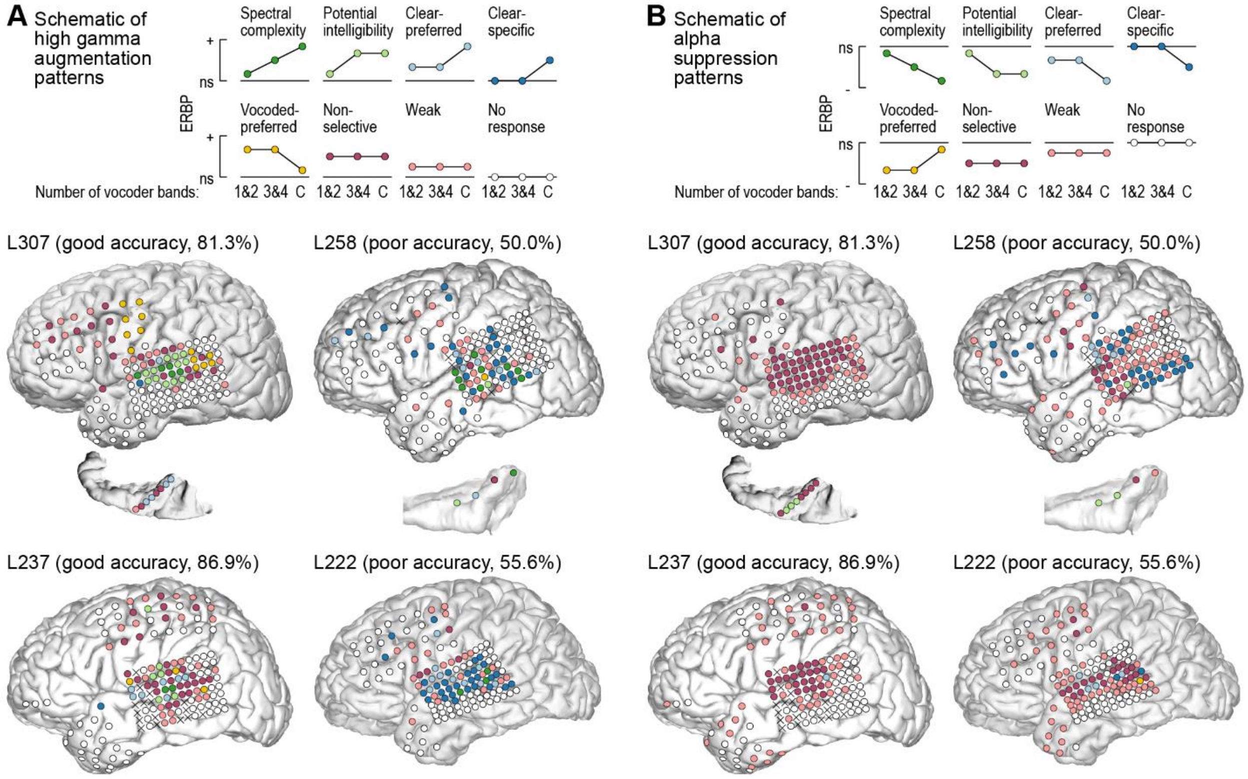

Supplementary Figure 4. High gamma augmentation **(A)** and alpha suppression **(B)** patterns elicited by noise-vocoded and clear speech in four exemplar participants (same as shown in **Figures 2** and **3**). Schematics of the eight patterns identified in the present study are depicted on top. Sites that were excluded from the analyses due to excessive noise or those confirmed to be seizure foci are depicted with an "×". **(A)** The most salient features that characterize the good performers (L307 and L237) are predilection for high gamma activity overlying the lateral STG to represent stimulus spectral complexity or its potential intelligibility, non-selective and vocoded-preferred responses which tended to cluster in STGP and SMG, and the overall relatively high prevalence of non-selective responses. This contrasts with the high prevalence of clear-specific high gamma responses in both poor performers (L258 and L222) over the lateral STG as well as in prefrontal cortex.

(B) Distribution of alpha suppression patterns in the same four participants. Here, the most striking differences between the good and poor performers is the high prevalence of non-selective suppression in good performers vs. clear-specific or weak suppression in the poor performers.

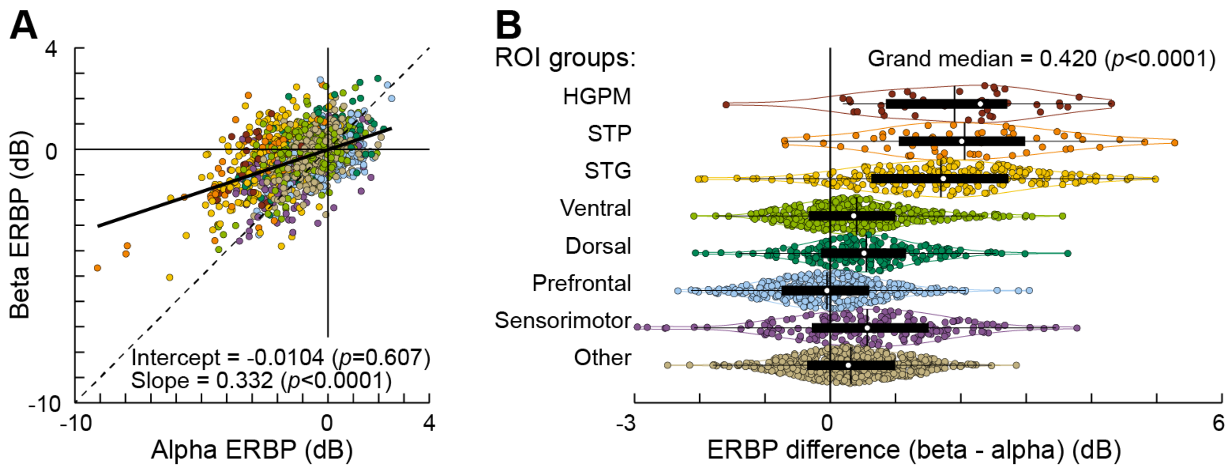

Supplementary Figure 5. Comparison between alpha and beta ERBP, measured in 250-500 ("early") and 500-750 ms ("late") time windows, respectively. Data from all recording sites, color coded by ROI group. **(A)** Linear regression analysis of alpha and beta ERBP. Thick black line denotes the regression line. **(B)** Pairwise differences between early beta and late alpha ERBP. Positive values indicate a greater degree of ERBP suppression if measured in the alpha compared to the beta band. In each violin plot, white circle denotes the median, vertical line denotes the mean, bar denotes Q_1 and Q_3, and whiskers show the range of lower and higher adjacent values (i.e., values within 1.5 interquartile ranges below Q_1 or above Q_3, respectively).

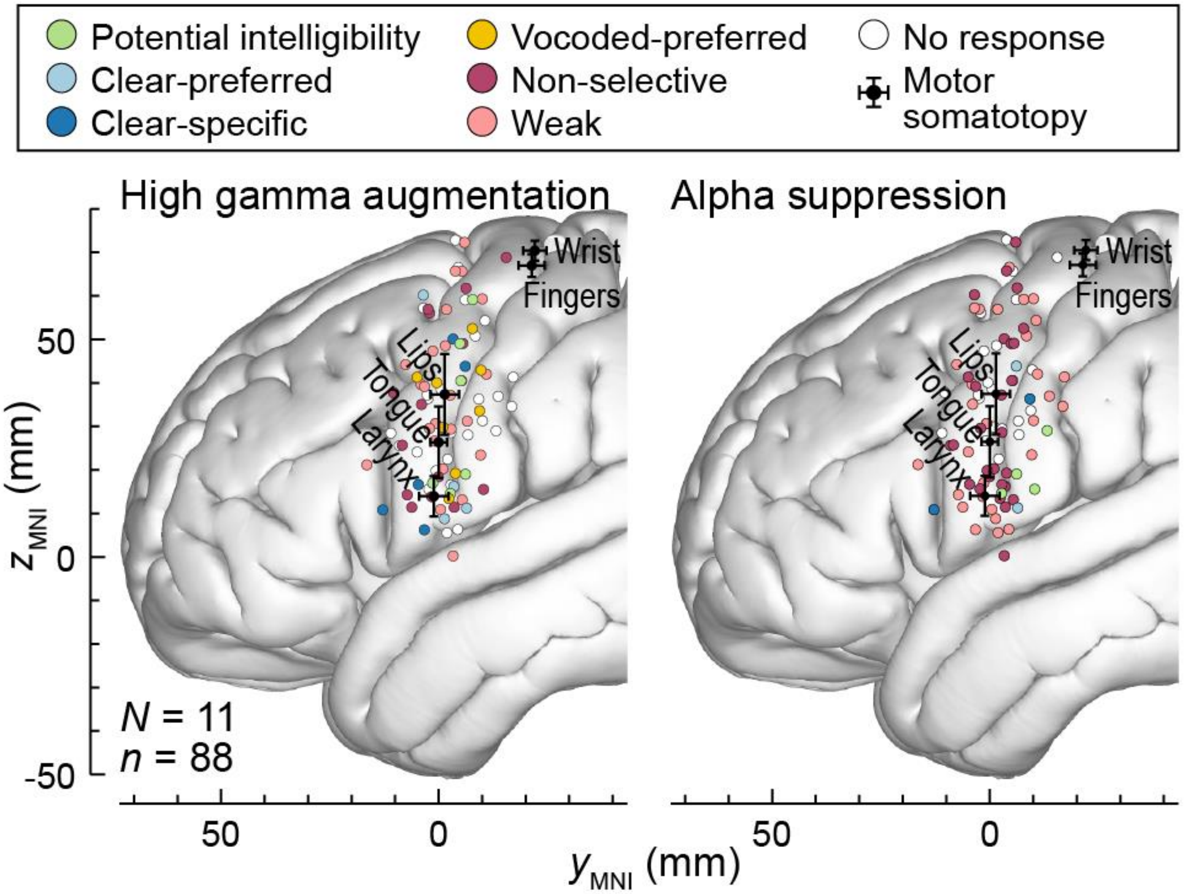

Supplementary Figure 6. Distribution of high gamma augmentation and alpha suppression (left and right panel, respectively) patterns in PreCG sites with respect to motor somatotopy. Summary data from 11 participants with PreCG coverage. Black symbols and error bars depict MNI coordinates of barycenters and their standard deviations of hand, wrist, fingers, lips, tongue, and larynx regions as reported by Roux et al. (2020). Each depicted recording site was confirmed to be located within PreCG based on each participant's individual anatomical reconstruction data. The apparent projection of some recording sites onto surrounding brain areas (e.g. PostCG) is a result of placing the electrode sites into the standard MNI coordinate system; the FreeSurfer average template brain is shown for spatial reference.

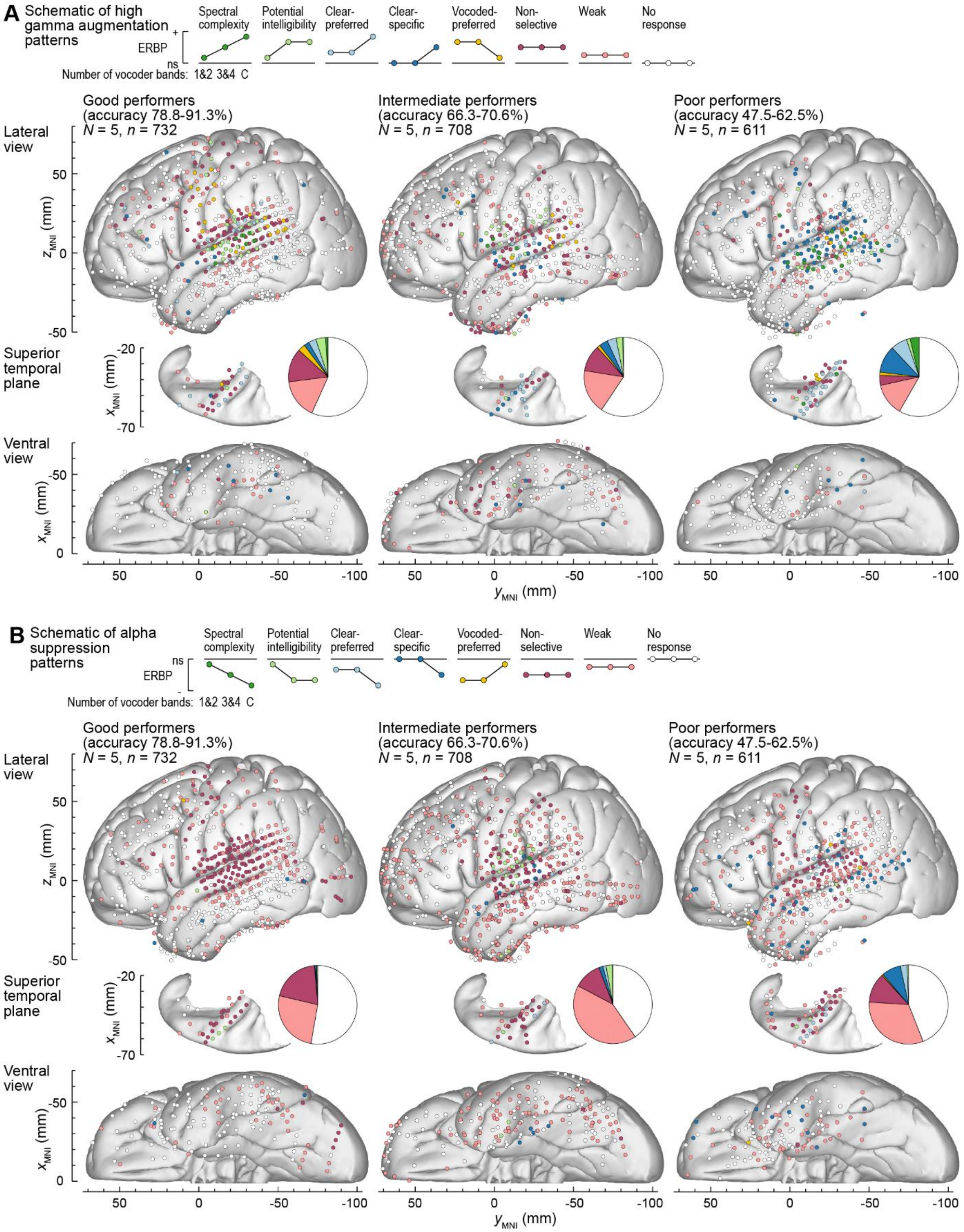

Supplementary Figure 7. High gamma augmentation **(A)** and alpha suppression **(B)** patterns in participants who exhibited good, intermediate, and poor performance in the behavioral task (left, middle, and right panels, respectively). **See caption of Figure 7 for details.**

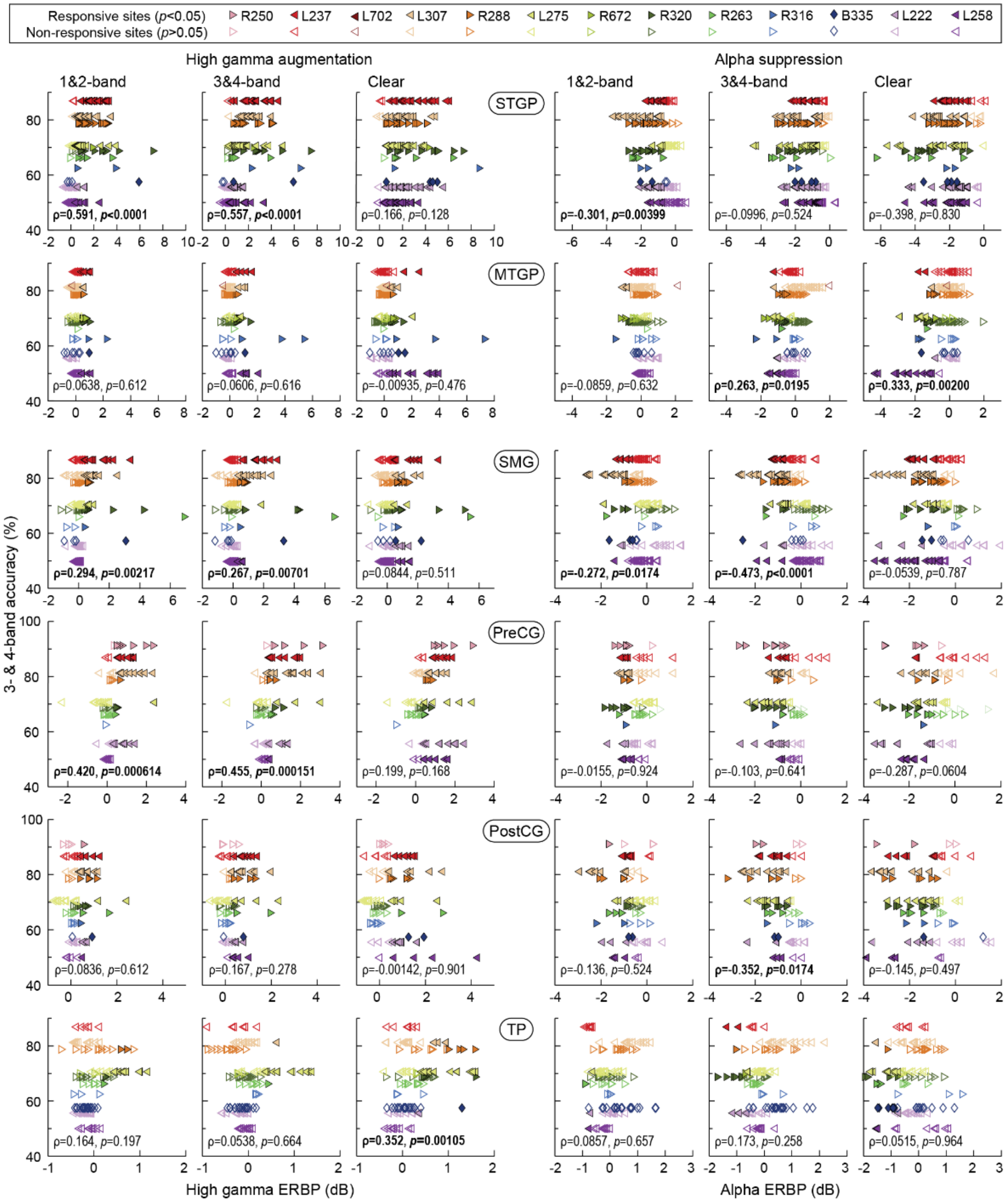

Supplementary Figure 8. Relationship between participants' cortical responses to vocoded and clear stimuli and average accuracy in the 3&4-band condition.

(caption continued on next page)

High gamma augmentation and alpha suppression are examined in the left and right panel, respectively. The three columns in each panel depict ERBP in response to 1&2-band, 3&4-band, and clear stimuli, respectively. The six depicted ROIs (STGP, MTGP, SMG, PreCG, PostCG, TP; rows) exhibited significant pattern distribution differences between good and poor performers (see **Figures 6B** and **7B**). Left- and right-pointing arrowhead symbols represent cortical sites in participants with mainly left- and right hemisphere electrode coverage, respectively. Participant B335 with bilateral electrode coverage is denoted by a diamond. Participants R322 and L282 did not have electrode coverage in any of the six depicted ROIs. Analyses were performed using Spearman's rank correlation with FDR correction for multiple comparisons. In STGM, Spearman's rank correlations did not reach significance (p<0.05, FDR-corrected) in any of the six comparisons (data not shown).

1.2 Supplementary Tables

Supplementary Table 1. High gamma augmentation patterns across ROIs.

ROI group	ROI	High gamma augmentation pattern (number of recording sites)								Total
		**Spectral complexity**	**Potential intelligibility**	**Clear-preferred**	**Clear-specific**	**Vocoded-preferred**	**Non-selective**	**Weak**	**No response**	
**Auditory core**	HGPM	1	0	21	0	2	16	0	0	40
**STP**	HGAL	0	2	14	6	0	11	6	0	39
	PP	0	0	2	1	1	3	0	6	13
	PT	1	1	3	1	0	0	0	0	6
**STG**	STGM	7	12	10	18	3	17	10	16	93
	STGP	15	18	20	23	11	40	21	6	154
**Ventral**	MTGA	0	0	0	1	0	1	2	34	38
	MTGM	0	0	0	0	0	4	13	61	78
	MTGP	4	2	2	7	1	10	15	106	147
	STGA	0	0	0	2	0	2	2	22	28
	STSL	0	0	0	0	0	4	10	35	49
	STSU	2	2	2	2	0	2	12	8	30
**Dorsal**	AGA	0	0	0	0	0	1	2	28	31
	AGP	0	0	0	0	1	0	0	11	12
	SMG	0	4	1	6	9	14	22	92	148
**Prefrontal**	ACC	0	0	0	0	0	0	2	3	5
	FMG	0	0	0	0	0	0	0	1	1
	IFGop	2	1	0	3	0	5	13	11	35
	IFGor	0	0	0	0	0	0	0	2	2
	IFGtr	1	2	1	1	0	2	7	31	45
	MFG	0	0	3	6	1	10	28	105	154
	OG	0	0	0	0	0	0	3	82	85
	SFG	0	0	0	1	0	0	4	26	31
	TFG	0	0	0	0	0	1	6	15	22
**Sensorimotor**	PostCG	1	2	4	4	0	16	19	39	85
	PreCG	0	6	4	5	8	15	24	26	88
**Other**	Amyg	0	0	0	0	0	2	13	24	39
	CingMA	0	0	0	0	0	0	0	1	1
	CingPD	0	0	0	0	0	0	0	3	3
	Cun	0	0	0	0	0	0	1	10	11
	FG	0	0	1	4	0	0	13	42	60
	fOperc	0	0	0	0	0	2	1	2	5
	GR	0	0	0	1	0	0	1	11	13
	Hipp	0	0	0	1	0	0	10	19	30
	InsA	0	0	0	0	0	0	2	8	10
	InsP	0	0	1	0	1	6	2	0	10
	IOG	0	0	0	0	0	1	6	6	13
	ITGA	0	2	0	2	0	3	7	38	52
	ITGM	0	0	0	1	0	2	4	44	51
	ITGP	0	0	0	3	0	1	6	20	30
	LingG	0	0	0	1	0	0	1	15	17
	MOG	0	0	0	0	0	2	4	34	40
	OP	0	0	0	0	0	0	0	5	5
	PHG	0	1	0	2	0	0	5	23	31
	PMC	0	1	0	2	1	1	9	19	33
	pOperc	0	1	0	0	0	0	0	0	1
	Precun	0	0	0	0	0	0	0	5	5
	SOG	0	0	0	0	0	0	1	5	6
	SPL	0	0	0	1	0	1	3	4	9
	TP	0	0	1	3	0	8	18	87	117

Supplementary Table 2. Alpha suppression patterns across ROIs.

ROI group	ROI	Alpha suppression pattern (number of recording sites)								Total
		Spectral complexity	Potential intelligibility	Clear-preferred	Clear-specific	Vocoded-preferred	Non-selective	Weak	No response	
**Auditory core**	HGPM	0	0	4	0	0	26	8	2	40
**STP**	HGAL	0	6	4	0	0	18	8	3	39
	PP	0	0	0	1	0	4	8	0	13
	PT	0	0	1	0	0	3	2	0	6
**STG**	STGM	0	6	2	2	0	46	27	10	93
	STGP	1	4	8	6	1	70	55	9	154
**Ventral**	MTGA	0	0	1	0	0	0	12	25	38
	MTGM	0	0	0	3	0	2	21	52	78
	MTGP	0	1	0	12	0	4	40	90	147
	STGA	0	0	1	2	0	1	18	6	28
	STSL	0	0	1	4	0	0	19	25	49
	STSU	0	1	0	0	0	2	14	13	30
**Dorsal**	AGA	0	0	0	1	0	1	5	24	31
	AGP	0	0	0	0	0	1	1	10	12
	SMG	0	0	0	6	0	27	60	55	148
**Prefrontal**	ACC	0	0	0	0	0	0	0	5	5
	FMG	0	0	0	0	0	0	0	1	1
	IFGop	0	1	0	2	0	7	13	12	35
	IFGor	0	0	0	0	0	0	0	2	2
	IFGtr	0	0	0	3	0	4	13	25	45
	MFG	0	0	0	1	0	2	43	108	154
	OG	0	0	0	2	1	0	16	66	85
	SFG	0	0	0	0	0	0	7	24	31
	TFG	0	0	0	0	0	0	9	13	22
**Sensorimotor**	PostCG	0	2	3	1	1	31	32	15	85
	PreCG	0	4	2	2	0	28	30	22	88
**Other**	Amyg	0	0	0	0	0	0	13	26	39
	CingMA	0	0	0	0	0	0	0	1	1
	CingPD	0	0	0	0	0	0	0	3	3
	Cun	0	0	0	0	0	0	3	8	11
	FG	0	0	0	4	0	1	31	24	60
	fOperc	0	0	0	0	0	1	3	1	5
	GR	0	0	0	0	0	1	3	9	13
	Hipp	0	0	0	0	0	1	7	22	30
	InsA	0	0	0	0	0	0	3	7	10
	InsP	0	0	0	0	0	2	5	3	10
	IOG	0	0	0	0	0	4	6	3	13
	ITGA	0	0	0	4	0	0	22	26	52
	ITGM	0	0	0	0	0	1	17	33	51
	ITGP	0	0	0	2	0	1	12	15	30
	LingG	0	0	0	0	0	1	8	8	17
	MOG	0	0	0	1	0	3	19	17	40
	OP	0	0	0	0	0	1	3	1	5
	PHG	0	0	0	2	0	1	7	21	31
	PMC	0	0	0	0	1	6	14	12	33
	pOperc	0	0	0	0	0	1	0	0	1
	Precun	0	0	0	0	0	0	0	5	5
	SOG	0	0	0	0	0	0	3	3	6
	SPL	0	0	0	0	0	0	5	4	9
	TP	0	2	0	3	0	0	40	72	117
